# Hopping or Jumping on the Cliffs: The Unusual Phylogeographical and Demographic Structure of an Extremely Narrow Endemic Mediterranean Plant

**DOI:** 10.3389/fpls.2021.737111

**Published:** 2021-11-10

**Authors:** Sandro Strumia, Annalisa Santangelo, Teresa Rosa Galise, Salvatore Cozzolino, Donata Cafasso

**Affiliations:** ^1^Department of Environmental, Biological and Pharmaceutical Sciences and Technologies, University of Campania “Luigi Vanvitelli”, Caserta, Italy; ^2^Department of Biology, University of Naples Federico II, Naples, Italy

**Keywords:** contemporary and historical gene flow, ddRAD, DIYABC, halophytes, IUCN, long distance dispersal, plant conservation, quaternary sea level oscillations

## Abstract

Several past and recent climatic and geological events have greatly influenced the current distribution of coastal species around the Mediterranean Basin. As a consequence, the reconstruction of the distributional history of these species is challenging. In this study, we used both chloroplast and nuclear SNPs to assess the levels of genetic differentiation, contemporary/historical levels of gene flow, and demographic history for the three only known (one mainland and two insular) populations of *Eokochia saxicola*, a rare Mediterranean coastal rocky halophyte. Plastid genome analysis revealed very low intraspecific haplotype variation and partial admixture among Capri and Palinuro populations with at least two independent colonization events for the Strombolicchio islet. Nuclear SNPs variation consistently identified three distinct genetic clusters corresponding to our sampling localities. Furthermore, strong genetic isolation was confirmed by both historical and contemporary levels of migration among the three populations. The DIYABC analysis identified two introductions temporally separated from Palinuro to Capri (ca.25 Mya) and subsequently to Strombolicchio (ca.09 Mya) as the most likely hypothesis for the current distribution of *E. saxicola*. Regardless of their small population sizes, all study sites supported high-genetic diversity maintained by outcrossing and random mating between individuals owing largely to wind pollination, an exclusive trait among Mediterranean narrow endemics. In conclusion, the patterns observed confirm that some Mediterranean endemics are not necessarily “evolutionary dead-ends” but rather represent species that have extensive demographic stability and a strong evolutionary legacy.

## Introduction

The Mediterranean Basin is one of the Hotspots of Biodiversity on the Earth (Médail and Quézel, 1999; Myers et al., 2000), largely due to the high number of endemic species (Quezel, 1985; Greuter, 1991). This biodiversity is the result of the current high heterogeneity of environmental matrix, climate, land uses, and geological and climatic events that occurred in the last 15 M years (Thompson, 2005; Blondel et al., 2010).

In particular, the most recent quaternary glacial-interglacial events have greatly influenced the current distribution of many plant species around the Mediterranean Basin (Médail and Diadema, 2009). The role of Glacial Refugia Areas and postglacial migration routes in shaping the current distribution of mainland European plants is well-known (Taberlet et al., 1998; Hewitt, 2001; Heuertz et al., 2004). However, the impact of glaciations on plant species mainly distributed on islands by climatic oscillations remains less explored. The Last Glacial Maximum (LGM) induced the lowering of sea levels, which subsequently led to the creation of several land bridges, facilitating the migration of plant species between the mainland and continental islands (Whittaker and Fernández-Palacios, 2007 quoting Wallace, 1880). Ultimately, the subsequent warming phases led to these temporary land bridges being gradually submerged by rising sea levels (Feliner, 2014). This main isolating barrier favored speciation processes, which resulted in the increase of insular endemics in the Mediterranean Basin (Médail, 2017). In contrast to insular endemics, water level oscillations played a different role in coastal halophytic species. Halophytes are salt-tolerant or salt-resistance species adapted to the very harsh habitat conditions due to high salinity (Ungar, 1991). These species are, therefore, typical of the coastal azonal vegetation [i.e., vegetation occurring where local ecological conditions, such as high salinity, overrule the effect of climate, according to Walter (1985)]. Indeed, for several coastal halophytes, the sea represents the main pathway for gene flow through the dispersal, even at long distances, of ramets or seeds (referred to as hydrochory) (Kadereit and Westberg, 2007; Westberg and Kadereit, 2009). Therefore, the current distribution of halophytic coastal species is the result of both past (main climatic oscillations and related sea-level variations) and recent abiotic factors (i.e., weather conditions, sea storms, and current sea circulations) acting on dispersion processes. Finally, the ecology of both species and plant communities can contribute to the complexity in the interpretation of coastal species distribution (Weising and Freitag, 2007; Feliner, 2014). The reconstruction of the distributional history of coastal halophytes is partly facilitated by their exclusive adaptations to hypersaline habitats that limit their distribution to coastlines, with a linear distribution range and patterns of long-distance dispersal being found to be more influenced by the direction of sea marine currents than by habitat filtering (Kadereit et al., 2005). However, the general pattern largely associated with coastal halophytes inhabiting dune and marshes habitats has not been found in coastal rocky species (Kadereit and Westberg, 2007). Both inland and coastal rocky areas provide suitable habitats for numerous endemic species often with extremely restricted distribution (Davis, 1951; Lavergne et al., 2004; Thompson, 2005) with many of them being considered “narrow endemics” (Kruckeberg and Rabinowitz, 1985; Médail and Baumel, 2018) and threatened by extinction (Montmollin and Strahm, 2005; Orsenigo et al., 2018). Therefore, they play a key role in the biodiversity conservation priority setting (Médail and Baumel, 2018).

*Eokochia saxicola* (Guss.) Freitag and G. Kadereith (Amaranthaceae) is a perennial evergreen shrub restricted entirely to coastal rocky environments exposed to the salt spray in very few locations around the South Tyrrhenian Sea (Strumia et al., 2020a). Historically, distribution records suggest *E. saxicola* occurred only on three small islands: Strombolicchio (a recent volcanic islet near Sicily), Capri (a calcareous island block), and Ischia (an old volcanic island, where it is now thought to be extinct). However, a new population has recently been discovered on the mainland coastal area along Campania and represents the only known mainland population of *E. saxicola* (Strumia et al., 2015). According to the criteria (less than 500 individuals today distributed in five or less populations) proposed by López-Pujol et al. (2013), *E. saxicola* can be considered an “extremely narrow endemic.” This species is a remnant of old lineages of Camphorosmae and is part of the *Chenolea* clade, originating in the early Miocene period during the evolution of the Mediterranean Basin (Kadereit and Freitag, 2011). To date, this clade includes only a few highly disjunct halophytic species, which are typical of warmer temperate climates. *E. saxicola* diverged from two other species [*Chenolea diffusa* Thunb. and *Spirobassia hirsuta* (L) Freitag and G. Kadereith] about 10.2 Mya (Kadereit and Freitag, 2011), i.e., largely before the onset of current Mediterranean climate (about 3.2 Mya, Suc, 1984) and is, therefore, considered a palaeoendemism (Favarger and Contandriopoulos, 1961; Stebbins and Major, 1965). Interestingly, *E. saxicola* distribution sharply contrasts with the general view that coastlines represent linear biogeographic systems connected by sea dispersion as reported for other coastal halophytes (Clausing et al., 2000; Kadereit et al., 2005). Genetic markers are ideal to study seed dispersal and show patterns of populations structuring determined by past and present gene flows (Bermingham and Moritz, 1998). This is particularly true in the case of species without pollen or fossil records. At the same time, the phylogeographical approach may also represent an important tool in conservation (Diniz-Filho et al., 2008) by identifying the populations that possess higher genetic diversity and, therefore, constituting a possible “genetic source” to be considered for future reintroductions (Médail and Baumel, 2018). The main aims of this study were, therefore, to answer the following questions: (1) Do populations of *E. saxicola* show any phylogeographic structure? (2) What are the primary factors that have shaped the phylogeographic structure? (3) What determines the genetic diversity and genetic structure of extant populations? (4) Can the combination of phylogeography and genetic diversity help define *E. saxicola* conservation priorities?

## Materials and Methods

### Study Species

*Eokochia saxicola* is a multi-branched perennial evergreen shrub found exclusively on maritime rocks (both calcareous and volcanic) close to the sea level in the area occupied by halophilic vegetation (Strumia et al., 2020b). Characteristic features of this species include protogyny, papillose stigma, and recalcitrant pollen, typical of wind pollinated plants (Barone Lumaga et al., 2016). Like other halophytes, the main seed dispersal mechanism is hypothesized to be hydrochory, owing mainly to the presence of diaspores able to float for several days. Moreover, a decrease in the germination rate of aging seeds and the inability for seedlings to anchor at suitable establishment sites have been assessed (Strumia et al., 2020a). According to IUCN, *E. saxicola* is threatened with extinction as it falls in the category *Endangered* (EN) (Santangelo et al., 2012; Orsenigo et al., 2018). The main threats are landslides in the few sites of occurrence and supposed reduction of fertility and reproduction success due to the small number of living individuals. We sampled *E. saxicola* in three locations representing the only known populations of its current distribution: Capri Island near Naples (Campania), Strombolicchio islet (Aeolian Archipelago, Sicily), and Palinuro, along the Cilento and Vallo di Diano and Alburni Nature Park coastline (Campania). Field collections were conducted from 2015 to 2017. Due to the inaccessibility of the cliffs (Strumia et al., 2020a), only the most accessible individuals were sampled. In each sampling site, the maximum estimate number of individuals was recorded through visual census (i.e., by binoculars from a boat) (Table 1).

**TABLE 1:** Sampling sites of *Eokochia saxicola* (abbreviation in brackets).

Site (Abbreviation)	Number of individuals	Sampled individuals
Capri – Grotta dell’Acqua (C)	120	18
Strombolicchio (K)	60	12
Palinuro Porto (P)	40	16
Palinuro Punta Iacco (PIK)	40	1
Palinuro Cala Fetente (CFK)	50	2
Palinuro-Camerota (CAM)	15	2
Total	325	51

*A maximum number of individuals (estimated at visual census) and a number of sampled individuals per site.*

### DNA Isolation, Library Preparation, and Sequencing

Genomic DNAs of 51 *Eokochia saxicola* individuals were extracted from preserved leaf material using the DNeasy Plant Mini Kit (Qiagen, Hilden, Germany), following the protocol of the manufacturer. The quantity and the purity of DNA extractions were checked using the NanoDrop ND 1000 Spectrophotometer (Thermo Fisher Scientific, Delaware), whereas the concentration was assessed with the Qubit fluorometer system (Invitrogen, United States) and the Quant-IT ds-DNA BR Assay kit (Invitrogen). Samples were sequenced using a slight modification to the standard ddRAD protocol (Peterson et al., 2012), with the restriction enzymes *Eco*RI and *Taq*I. A single library with barcoded individuals was sequenced in a single Illumina HiSeq 2500 lane for 150-base single-end reads. From a subset of samples (16 *E. saxicola* + *S. hirsuta*), a “genome skim” was performed (Straub et al., 2012) to gather sufficient data for complete plastid assembly without prior enrichment or isolation of plastid DNA (Coissac et al., 2016; Supplementary Table SM1). Libraries were constructed according to the Nextera DNA Library Prep Kit (Illumina) protocol and Nextera XT indexes (Kit v2 Set A), which were used to multiplex the individual samples. Libraries were sequenced in a single Illumina HiSeq 2500 lane for 150-base single-end reads.

### Preprocessing and Analysis of the Complete Plastome Sequences

The *Eokochia saxicola* and *Spirobassia hirsuta* raw sequence reads were quality checked with the FastQC v0.11.9^1^, and low-quality reads and adapters were subsequently trimmed with Trimmomatic v0.39 (Bolger et al., 2014). We implemented a *de novo* assembly of *S. hirsuta* plastome by setting kmer = 121 in Velvet v1.2.10 software (Zerbino and Birney, 2008), and trimmed reads were then further assembled using Pilon v1.23 (Walker et al., 2014). We implemented FASTQ Screen v0.14 (Wingett and Andrews, 2018) for filtering the plastid reads from each *E. saxicola* individual by using a *de novo*-assembled *S. hirsuta* plastome (unpublished), and orphan reads were removed through the python script FASTQ Combine Paired End.py^2^. The *E. saxicola* filtered reads were mapped to *S. hirsuta* plastome by using BOWTIE2 v2.3.5.1 (Li and Durbin, 2009). The alignments (BAM files) produced a single-variant call format (vcf) file for all samples, where the SNPs were identified using bcftools v.1.9 with the setting “mpileup -Ou” and called *via* bcftools using the −mv and –ploidy 1 functions. The vcf file was converted to a Nexus format, and a haplotype network (also including available *Amarantaceae* plastome sequences) was built by using the TCS method implemented in PopArt v1.7 (Leigh and Bryant, 2015).

### Preprocessing ddRAD Data

Raw reads were demultiplexed in individual paired-end libraries and filtered for low-quality reads with the process_radtags pipeline in STACKS v2.4 (Rochette et al., 2019). Quality control of demultiplexed reads was performed with FastQC v0.11.9. The catalog RAD-loci was built with *de novo* assembly using the *de novo*_map.pl wrapper in STACKS v2.4 by using parameters *m* = 1, *M* = 8, and *n* = 1, following the optimization procedure described in Paris et al. (2017). The population program in a Denovo_map.pl wrapper was used for collecting the polymorphic SNPs in a vcf file. The vcf files (with 48 individuals) were analyzed with the –missing function of PLINK v1.07 (Purcell et al., 2007) for calculating the percentage of missing data (approximately ∼81% of missing data). Lastly, to avoid the risk of calling false SNPs, we reduced the dataset to include all sites with a minor allele frequency (MAF) greater than or equal to 0.05 and with a (–*max*-*obs*-*het)* setting of 0.70 (as also suggested in Gargiulo et al., 2021). Finally, to account for the effects of missing data per individual, VCFTOOLS v0.1.14 (Danecek et al., 2011) was implemented to filter the minimum number of ddRAD loci shared by at least 70% of individuals (i.e., the full dataset). To minimize the potential effect of missing data in altering the results, some population demographic analyses were also or only performed on a subsample of individuals by maximizing the number of reads per sample and minimizing the amount of missing data. Therefore, a reduced dataset was constructed with 25 individuals (i.e., only those ranging between 2,000,000 and 500,000 reads, namely 10 individuals from Palinuro, eight from Capri, seven from Strombolicchio, see Supplementary Text 1.1), which were filtered so that missing data were present in two or less individuals for each locus (i.e., ddRAD loci present in 95% of the individuals).

### Analysis of ddRAD Data

#### Analyses on the Full (48 Individuals) and Reduced Dataset (25 Individuals)

Maximum Likelihood Tree (ML) trees were generated from both datasets. GTRCAT was used as the substitution model for nucleotide sequences, as implemented in RAxML v8.2.12 (Stamatakis, 2014). The resulting trees were drawn to scale with Figtree v1.4.4^3^.

The R package AWclust (Gao and Starmer, 2008) was implemented as a non-parametric distance-based clustering model (allele-shared distance) for the analysis of populations structure with a HapMap format file as input. We estimated the best K using 100 simulations with an interval of K from 1 to 6. To visualize the genetic relationships among individuals, we performed a multidimensional scaling (MDS) based on the optimal number of clusters (best K).

The vcf file (48 individuals) converted into a genind object with the function vcfR2genlight in the R package adegenet v2.02. (Jombart, 2008) was subsequently used for the Discriminant Analysis of Principal Components (DAPC) (Supplementary Text 1.2 and Supplementary Figures 5–7). Lastly, the optimal number of clusters (*K*) was then determined by the lowest Bayesian Information Criterion (BIC), and the DAPC plot was constructed with the function scatter.dapc.

Pairwise Fst among individuals and populations was calculated with the function stamppFst (in the R package adegenet), and the result was visualized in a heatmap plot with function heatmap 0.2. The fineRAD structure was implemented to infer population structure *via* shared ancestry in the ddRAD dataset, focusing on the most recent coalescence (common ancestry) among the sampled individuals (Malinsky et al., 2018). Samples were assigned to populations using 100,000 iterations as burn-in before sampling 100,000 iterations. The trees were built using 10,000 iterations and the output was visualized using fineStructure GUI.

#### Analyses on the Reduced Dataset (25 Individuals)

The following analyses, more sensitive to the excess of missing data, were conducted with the reduced dataset containing 25 individuals and filtered for a minimum number of ddRAD loci shared by 95% of individuals. The function basic.stats in R package hierfstat (Goudet, 2005) was implemented for the estimation of observed heterozygosity (Ho), expected heterozygosity (He), and the within-population inbreeding coefficient (Fis).

We focused on the timing of the connectivity change based on the estimates of historical and contemporary migration by using a coalescent-based method (MIGRATE) and a disequilibrium-based method (BayesAss), respectively. The historical migration rate (Supplementary Text 1.4 and Supplementary Table SM3) was estimated using Bayesian inference in MIGRATE (Beerli, 2006), while the contemporary migration rates (Supplementary Text 1.3; Supplementary Table SM2; Supplementary Figures 8, 9) were estimated from the current generation and two past generations using Bayesian inference in BayesAss v3 (Rannala, 2007).

Alternative scenarios of origin, the direction of migration, and relationships among *E. saxicola* populations were tested on the ddRAD dataset under the coalescent-based approximate Bayesian computation in DIYABC Random Forest v1.0 (Collin et al., 2021). This enables the comparison of different population demographic models and determines the estimation of their parameters without calculating complex likelihood functions (Beaumont, 2010). Different historical demographic scenarios were tested with the following Ne-prior parameters set for each population: Npalinuro (100–500); Ncapri (10–500) and NStrombolicchio (10–50). This low value of Ne priors is based on the rarity of *E. saxicola* among the different locations (Table 1).

For *Eokochia saxicola*, a generation time of 10 years was considered (*ex situ* cultivated 5-year-old plants have not bloomed yet). Then, in accordance with the geological age of the sites-hosting current populations (Vezzoli, 1988; Gillot and Keller, 1993; Santangelo et al., 2011), we set the split time (t) as a number of generations from 10 to 50,000. Conditions were set as t1 > t2, according to the timing of events. Bottleneck priors (db time) were set from 10 to 100 generations and the number of founders (Nb) from 10 to 50 individuals. Since previous analyses based on genetic structure did not show admixture events, we did not include admixture events into scenarios.

Four scenarios (Supplementary Figure 10) were designed to explore alternative hypotheses regarding the origin and mainland-island migrations (Scenario 1). The colonization of Capri occurred through a long-distance dispersal event of a small fraction of the Palinuro population at time t1. Then, the bottleneck event in the newly founded population (i.e., Capri) was modeled through a limited number of founders (Nb) during a short period (t1-db). At time t2, the Strombolicchio population was born through a long-distance dispersal event of a small fraction of the Capri population. Then, the bottleneck event in the newly founded population (i.e., Strombolicchio) was modeled through a limited number of founders (Nb) during a short period (t2-db) (Scenario 2). The colonization of Capri occurred through a long-distance dispersal event of a small fraction of the Palinuro population at time t1. Then, the bottleneck event in the newly founded population (i.e., Capri) was modeled through a limited number of founders (Nb) during a short period (t1-db). At time t2, the Strombolicchio population was born through a long-distance dispersal event of a small fraction of the Palinuro population. Then, the bottleneck event in the newly founded population (i.e., Strombolicchio) was modeled through a limited number of founders (Nb) during a short period (t2-db) (Scenario 3). An ancestral population split between Capri and Palinuro populations occurred at time t1. At time t2, Strombolicchio colonization occurred through a long-distance dispersal event of a small fraction of the Capri population. Then, the bottleneck event in the newly founded population (i.e., Strombolicchio) was modeled through a limited number of founders (Nb) during a short period (t2-db) (Scenario 4). An ancestral population split between Capri and Palinuro populations occurred at time t1. At time t2, Strombolicchio colonization occurred through a long-distance dispersal event of a small fraction of the Palinuro population. Then, the bottleneck event in the newly founded population (i.e., Strombolicchio) was modeled through a limited number of founders (Nb) during a short period (t2-db) (Supplementary Figure 10 and figure caption).

We processed 1,000,000 simulations datasets, ∼250,000 simulations datasets per scenario. For scenario choice, a linear discriminant analysis (LDA) processed on the summary statistics was used before processing Random Forests (RF) predictions (Supplementary Figure 11). The 250,000 simulation datasets (i.e., higher than the suggested 20,000 simulation datasets) of the best supported scenario were used to evaluate the model choice and parameter estimation in RF analysis. The number of trees in the constructed random forests were fixed to *n* = 1,500, the minimum number to ensure a stable accuracy measure of the best scenario and to estimate the mean value and the lower and upper 95% quartiles of the posterior distributions (Chapuis et al., 2020; Supplementary Figure 12).

## Results

**FIGURE 1:**
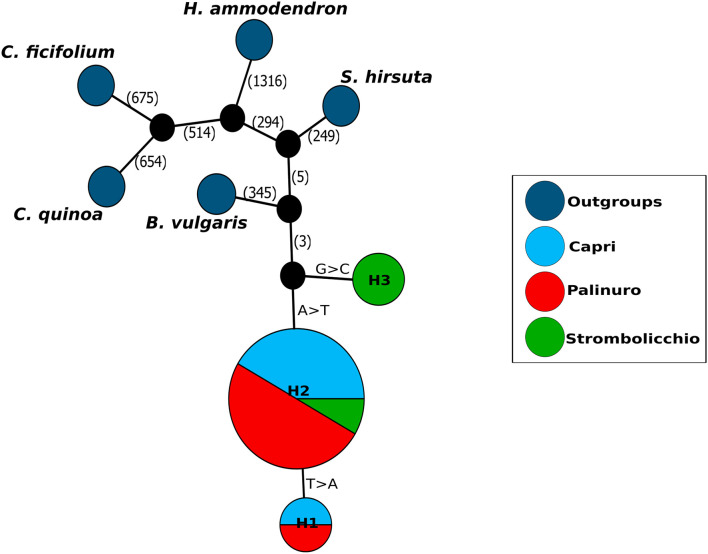
Plastid haplotype network. Each circle represents a different haplotype, where circle size is proportional to the number of individuals that carry the same haplotype. Black circles indicate missing intermediate haplotypes. Mutational steps are reported on the lines of the haplotype networks. Colors show the genotypes belonging to different geographic origins.

For the Illumina sequencing for the analysis of SNPs variation in the complete plastome sequences of 16 *E. saxicola* + *S. hirsuta*, 41,187,562 raw reads (150 bp for average read length) were generated; of which, 34,528,403 reads remained after performing the preprocessing procedure (Supplementary Text 1.1 and Supplementary Table SM1). Variant calling revealed 333 SNPs among both *S. hirsuta* and *E. saxicola* individuals as opposed to only three SNPs detected among *E. saxicola* individuals. The location, nucleotide change, annotation, and mutation type of these three SNPs in *E. saxicola* are provided in Supplementary Table 1. These SNPs identified three haplotypes in the *E. saxicola* populations, with haplotype H2 present in all three locations and haplotype H1 shared between the mainland and Capri Island, whereas the haplotype H3 was exclusive to Strombolicchio islet (Figure 1; Supplementary Table 1). All other Amarantaceae plastomes, including the newly assembled *S. hirsuta*, complete plastome (data not shown) differed largely from *E. saxicola* plastome both in size and number of SNPs.

From ddRAD sequencing, a total of 30,022,310 raw reads were obtained. After which, 29,276,067 reads were identified through de-multiplexing and filtering using STACKS pipeline to remove any low-quality read, ambiguous barcodes, and sequences without cut sites. We obtained, on average, 609,918 raw reads per sample and 224,637 mapped reads per sample (Supplementary Table SM1).

The pipeline *de novo*_map.pl generated 6,476,170 loci and assembled ∼ 99.9% of loci into contigs. The total length of pair-end contigs (the generated *de novo* assembly) was approximately 175 Mbp, as calculated with the software Bandage (Wick et al., 2015). The average mapping efficiency of our samples to this *de novo* assembly was two reads per locus, as calculated with module gstack of the Stack pipeline. In total, 10,782,592 reads were aligned with the generated genome, i.e., 70% of raw reads did not align (i.e., they were unmapped unique reads). Due to this low coverage, SNPs were called by adopting a variant calling strategy of 3×-minimum coverage. With our filtering approach, in the 48 individuals/3,962 SNPs datasets, we never observed any case of multiple variants in the same locus (suggestive of a diploid status for *E. saxicola*) or of variants called in less than five individuals. With this conservative approach, we may have lost some rare variants, but we have reduced the risk of calling false SNPs due to low coverage.

An ML tree was built with 3,962 SNPs present in at least 70% of samples. The 48 individuals clustered distinctly in three separate clades. The separation of three distinct clades was further emphasized when using the reduced dataset of 25 individuals with 120 SNPs shared by 95% of individuals (Supplementary Figure 1).

**FIGURE 2:**
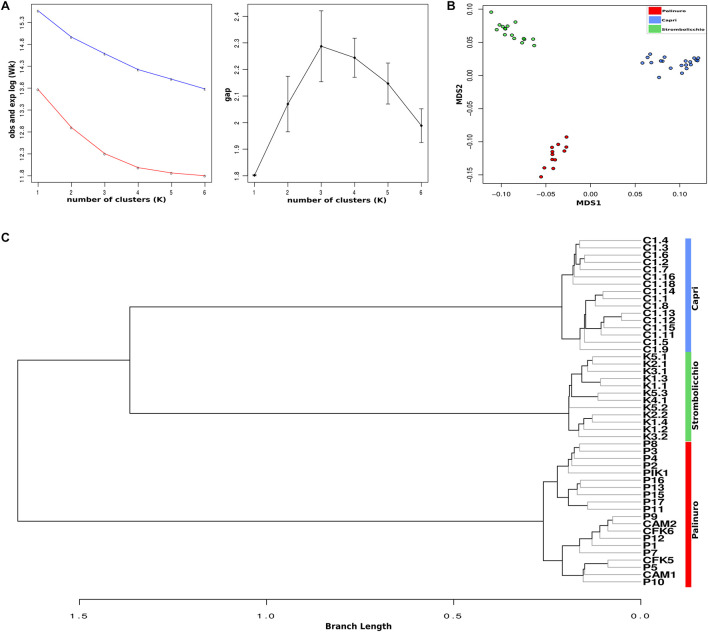
Genetic clustering of sampled individuals. **(A)** Left: The red line represents the log of the pooled within-cluster sum of squares (Wk) from the observed data. The blue line represents the expected log of Wk from a uniform distribution. Right: the actual gap statistic. **(B)** A clustering by size reduction (an MDS plot) is represented. Individuals are distributed as points in a two-dimensional vector space. **(C)** The ancestry-inferred tree with AWclust.

Genetic Clustering with Awclust software identified *K* = 3 as the optimal number, with each cluster corresponding to a population (Figure 2A). The 48 individuals were split into three distinct clusters or geographical regions in the MDS analysis too (Figure 2B). Moreover, the AWclust dendrogram plot displays a clade with two clusters (the island populations) and a clade with the mainland Palinuro population (Figure 2C).

**FIGURE 3:**
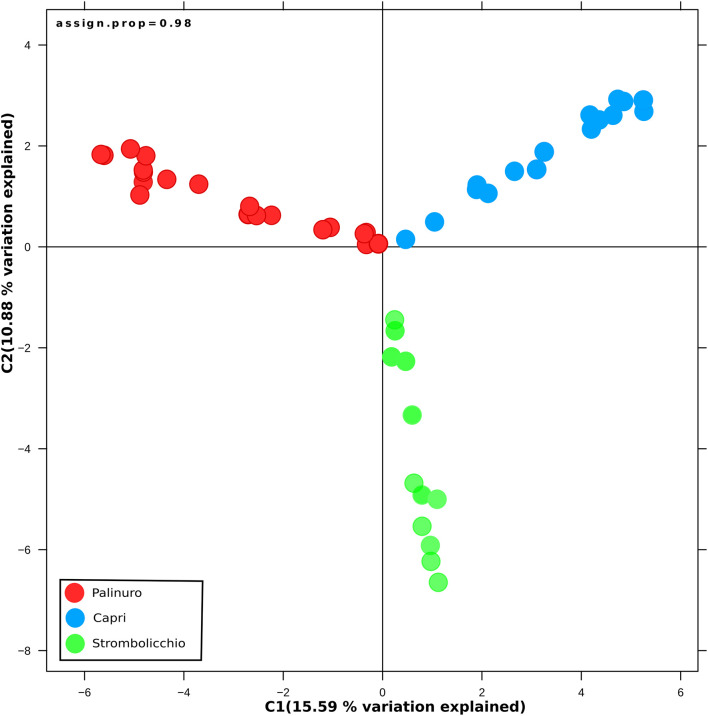
Discriminate analysis of principal components (DAPC). The analysis was drawn using 3,962 SNPs across 48 individuals (SNPs present in at least 70% individuals) and was constructed using 20 principal components (PCs) and two discriminate functions. Dots represent individuals, with colors denoting sampling origin.

Visualization of population structure using a DAPC (Discriminant Analysis of Principal Components) concordantly revealed three distinct genetic clusters, which included the two island populations (Strombolicchio and Capri) and the mainland Palinuro population (Figure 3; Supplementary Figure 7).

**FIGURE 4:**
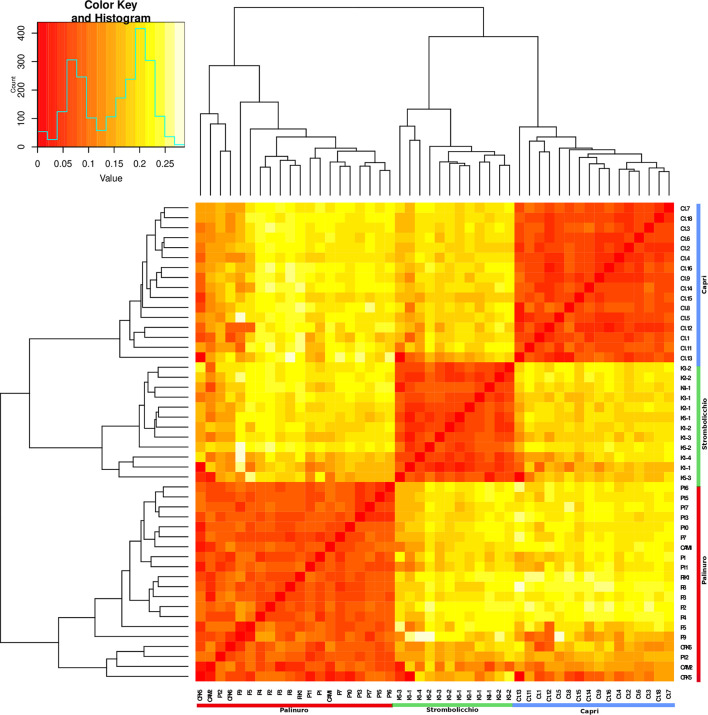
A heatmap graph of Fst was built by using 3,962 SNPs across 48 individuals (SNPs present in at least 70% of individuals). The heatmap illustrates the Fst pairwise matrix based on a color code: orange/red color (low Fst) to white (high Fst). Along the axes of the matrix, phylogenetic trees are constructed using the Neighbor Joining method. The legend (top left) depicts the color-coded Fst values.

**FIGURE 5:**
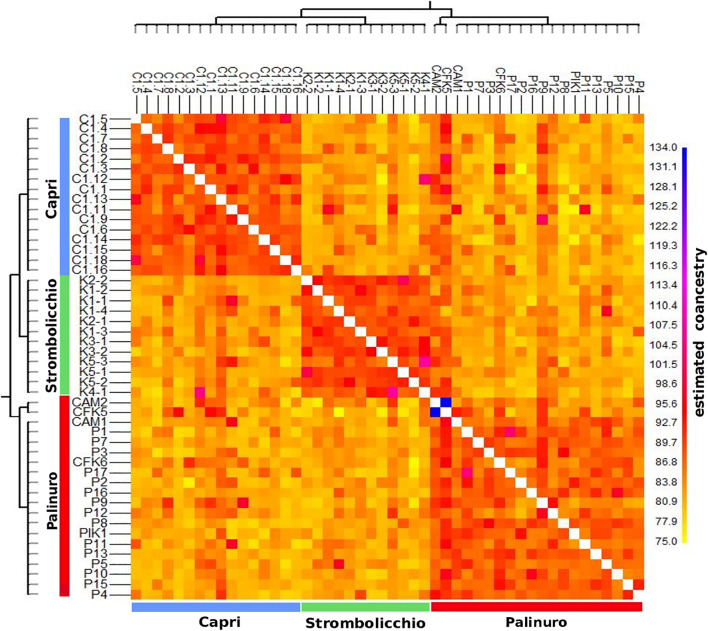
The co-ancestry matrix was shared among 48 individuals (SNPs present in at least 70% individuals). The co-ancestry is calculated as the number of different sequences (i.e., SNPs) between pairs of individuals. The color of each cell in the matrix indicates the expected number of chunks imported from a donor genome (horizontal axis) into a recipient genome (vertical axis). Therefore, highly different cells are indicated in yellow, while indistinguishable cells are represented by blue/purple. Lastly, two phylogenetic trees with the Neighbor-Joining method are represented along the *x* and *y* axes.

The heatmap plot confirmed the existence of the three populations (Figure 4). Overall, all populations revealed a moderate level of intrapopulation co-ancestry (Figure 5). Comparable results were gathered with the reduced dataset (by only using 120 SNPs present in at least 95% individuals across 25 individuals) but with a higher intrapopulation co-ancestry value as it was found inversely correlated with a number of SNPs and missing data (Supplementary Text 1.1; Supplementary Figure 4).

The mean genetic diversity parameters among populations were 0.0941 for expected Heterozygosity (He), 0.0841 for observed Heterozygosity (Ho), and 0.0781 for the Fixation index (Fis), respectively. Interestingly, the Palinuro population had the highest inbreeding value (Fis = 0.2343) (Table 2).

**TABLE 2:** Estimates of genetic diversity parameters over 25 individuals (SNPs present in at least 95% individuals) implemented with function basic.stats in the hierfstat R package.

Population	Ho	He	Fis
Capri	0.0982	0.1071	0.0833
Strombolicchio	0.0612	0.0704	0.1307
Palinuro	0.0929	0.1048	0.2343
Mean	0.0841	0.0941	0.0781

**FIGURE 6:**
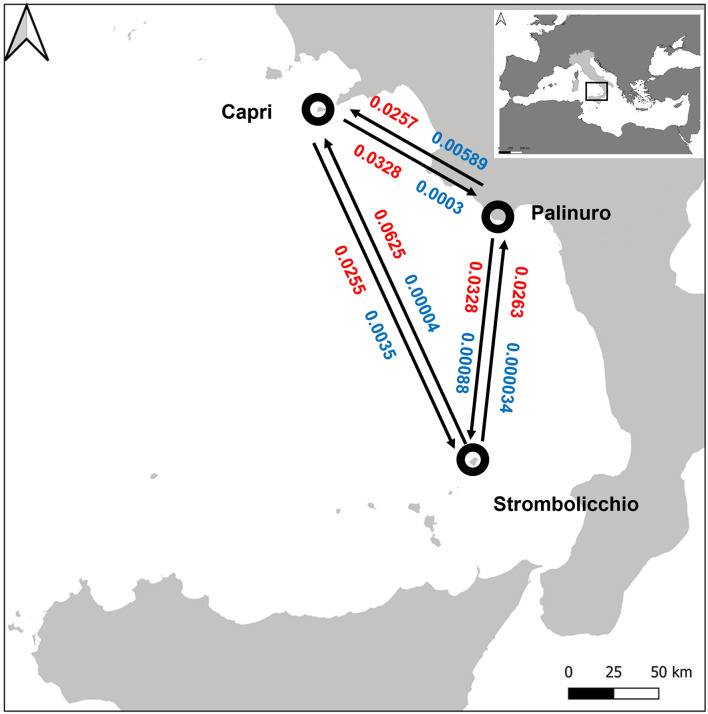
Estimates of the mean posterior recent (blue) and historical (red) migration rate among 25 individuals (SNPs present in at least 95% individuals). The circles represent populations and arrows indicate the direction of migrant individuals between different populations. The inset plot at the top right of the figure reports the position of the map in the Mediterranean Basin.

Multiple runs of BAYESASS yielded low levels of contemporary gene flow (mc, fraction of individuals that are immigrants) among the three populations. Concordantly, also, the historical rates of migration as calculated in MIGRATE were generally very low (Figure 6; Supplementary Table 2). Low mc and mh values (mh lower 95% CI includes zero) suggest that populations have become demographically independent in the past. Despite the reduced geographic distance, historical rates of migration between Palinuro and Strombolicchio have the lowest values. Some hidden environmental barriers (likely North heading sea currents) may have impeded any past migration between these two populations.

**FIGURE 7:**
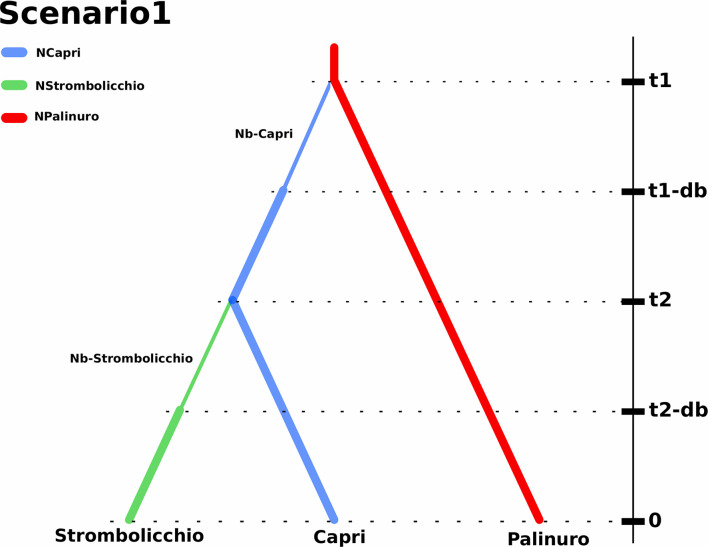
Demographic history of *Eokochia saxicola* populations implemented by DIY ABC. Scenario 1 represents the most likely hypotheses of the demographic history: the Strombolicchio population was derived from the Capri population, which originated from the Palinuro population. The branch colors indicate discrete population size parameters in the model. t1 represents the split time between the Palinuro population and Capri population, while t2 represents the split time between the Capri population and Strombolicchio population. The thin branch width indicates bottlenecks of duration db (t1-db and t2-db) with effective population sizes of Nb (Nb-Capri and Nb-Strombolicchio). Note: Time is not to scale.

The *E. saxicola* dispersion was inferred to have occurred by temporally separated introductions from the Palinuro to Capri and by the latter, to Strombolicchio as revealed by DIYABC model selection, scenario 1 (Figure 7). This scenario was supported by the model (votes = 781; posterior probability *p* = 0.501). The alternative scenario (2) where Strombolicchio originated from Palinuro received 231 votes. Instead, the scenarios (3 and 4) suggesting the split of an ancestral population between Capri and Palinuro and the subsequent colonization of Strombolicchio from Capri or Palinuro received 308 and 180 of the votes, respectively.

The colonization of Capri from the mainland population was predicted to have occurred around 24,615 generations ago while the following colonization of Strombolicchio only occurred 9,003 generations ago. Interestingly, the relatively narrow posterior parameter distribution of the population introduction from the mainland Palinuro to the Capri Island suggests this single event was followed by a bottleneck period (db) of at least 43 generations with a relatively small founding population of 10 individuals. The bottleneck size of the Strombolicchio population (Nb-Strombolicchio) was around nine individuals. However, at time 0, the effective population size of Strombolicchio is 32 individuals, while the effective population size of Capri is 369 (249–484), i.e., comparable with 379 (150–483) of Palinuro (Table 3).

**TABLE 3:** Prior values (minimum and maximum, with uniform distribution) for the parameters used for the demographic scenarios and posterior values (mean; median; quantile 5%, and 95% and variance) were estimated from Scenario 1 in the DIYABC approach.

	Parameter	Prior parameters		Posterior parameters				
			
		minimum	Maximum	mean	median	quantile 5%	quantile 95%	variance
Effective population size	Ncapri	10	500	369	371.25	249	484	9242.41
	NStrombolicchio	10	50	32	30.9	15	46	1053.76
	NPalinuro	100	500	379	381.03	150	483	4228.4
	N1b	5	50	10	8	3	18	421.353
	N2b	5	50	9	924.378	2	18	317.939
Time scale in generations	t1	10	50.000	24615	13035	17217.3	47268	8.22E + 07
	t2	10	50.000	9003	10096.1	4261	30256	3.98E + 07
	db	10	100	43	37	12	96	1446.28

## Discussion

The integration of different nuclear and plastid genetic markers provided novel insight into the temporal and spatial distribution, a colonization pathway, and dispersal patterns of *E. saxicola*. In particular, both markers contributed to disclosing the roles of contemporary versus historical processes in shaping the current genetic variation of *E. saxicola* populations. Despite its paleoendemic status, the present distribution and partition of genetic diversity in *E. saxicola* revealed a rather recent dispersion/fragmentation, most likely occurring during the last quaternary climatic oscillations, which resulted in the almost complete interruption of ongoing gene flow among living populations.

It is well-known that different filtering strategies influence both the estimation of genetic diversity and differentiation (Cozzolino et al., 2020; Gargiulo et al., 2021). Therefore, for testing the robustness and reliability of our results using the ML tree, pairwise Fst and co-ancestry among individuals and populations were calculated both with the full dataset (3,962 SNPs across 48 individuals, SNPs present in at least 70% individuals) and with the reduced dataset (25 individuals by selecting 120 SNPs shared by at least 95% of individuals). We found largely overlapping results between the two datasets (Supplementary Figures 1–3). Still, as expected, when using different stringency criteria in loci selection, when comparing datasets with SNPs present in 70% vs. 95% of the samples, we found a decrease of between-lineage differentiation of the employed loci as measured by global *F*_*ST*_ and an increase in the average overall estimated co-ancestry (Supplementary Text 1.1. and Supplementary Figure 4). Thus, our conservative approach may have not precisely estimated the values of some population genetic parameters (e.g., heterozygosity, *F*_*ST*_, Ne, and migration rates).

### Do Populations Show Any Phylogeographic Structure?

Analysis of full plastid genome variation in *E. saxicola* revealed a surprisingly low level of intraspecific variation (only three haplotypes differing for a maximum of two mutation steps) among the three isolated populations with a considerable number of differences (in total plastome length too) with the related *S. hirsuta*. Since large interspecific differences can be expected between paleoendemic species separated approximately 10 Mya (cfr. Kadereit and Freitag, 2011), both haplotype admixture in populations and very low haplotype variation at the intraspecific level support a recent separation of current *E. saxicola* populations. The Palinuro and Capri populations share two haplotypes (H1 and H2), differing by a single-base mutation (Figure 1). The most common haplotype (H2) was found in the Strombolicchio population, but, here, the “distant” haplotype H3 (i.e., differing by two mutational steps) was also detected (Figure 1). Even if the scenario of dispersal from Palinuro is the best supported, actual co-occurrence of two related haplotypes in both insular Capri and mainland Palinuro does not rule out an alternative scenario where these populations were likely part of a larger, ancestral population that later split into today isolated populations without a bottleneck phase. Interestingly, this scenario is the second-best supported scenario (i.e., Scenario 3) in the DYABC analysis based on nuclear markers (discussed below).

### What Are the Primary Factors That Have Shaped the Phylogeographic Structure?

According to the morpho-bathymetric reconstruction of the Southern Tyrrhenian Sea (Aiello et al., 2015) and considering that, during the last glacial phases, the sea level decreased up to 100 meters, Capri was once connected to the mainland cliffs of Southern Italy. Moreover, the coastline shape between Capri and Palinuro drastically changed during the Quaternary due to the combination of tectonics, sedimentary inputs, volcanism, and sea-level oscillations (Santangelo et al., 2017). In this scenario, which occurred in the last 1.8 Mya, both frequency and surface extension of unsuitable (defined as sandy coasts related to alluvial plains) and suitable (defined as rocky coasts related to pre-quaternary geological substrates) habitats for *E. saxicola* were transformed from the sea-level oscillations. Moreover, these oscillations most likely reduced the “jumping” distance from the Palinuro cliffs to Capri, favoring a linear “hopping” migration along the coastline, as also reported for several other Mediterranean halophytes (Clausing et al., 2000; Kadereit et al., 2005; Weising and Freitag, 2007). The current distribution of *E. saxicola* along the Palinuro cliffs (ca. < 15 km) further supports the evidence of a “hopping” dispersal ability along with these coastal rocky habitats.

In contrast, the relatively young volcanic islet of Strombolicchio (0.2 Mya) has been completed isolated from any mainland, even during the Quaternary seal-level oscillations. Accordingly, long distance dispersal or “jumping” is the only plausible scenario for the colonization of *E. saxicola* on Strombolicchio. Surprisingly, despite the rarity of *E. saxicola* populations and the small size of the islet, at least two occasional events (two different plastid haplotypes) of colonization likely occurred as founder events for the Strombolicchio population (Figure 1). Hydrochory, as for many other halophytes of the Mediterranean Sea (Kadereit et al., 2005; Arafeh and Kadereit, 2006; Weising and Freitag, 2007; De Castro et al., 2020), may have contributed to the long-distance dispersal of *E. saxicola* seeds. Indeed, sea-drifted seeds depend entirely on currents for their dispersal, and there is some evidence from the surface circulation observed in the southern Tyrrhenian Sea (Vetrano et al., 2004; Rinaldi et al., 2010) that is consistent with the known distribution of *E. saxicola*. Changes in sea currents due to the presence of a vortex north of Capri renders the north and south Tyrrhenian Sea “isolated” from each other (Procaccini et al., 2001) and impedes seeds dispersal toward the north.

According to the average reported speed of south Tyrrhenian surface circulation (Rinaldi et al., 2010), the floating time ability of the *E. saxicola* (Barone Lumaga et al., 2016) would have been sufficient to reach Strombolicchio islet from the Palinuro/Capri coastlines. Haplotype admixture between Capri and Palinuro does not allow detecting the source population, but nuclear data point to the Capri population as more proximate to the Strombolicchio one. Nevertheless, the presence of a second haplotype in the young islet, assuming that this haplotype may not have locally evolved or that it was present in Palinuro/Capri and gone undetected/extinct, points to a second colonization event from an unknown source population. This latter hypothesis is supported by the distinctiveness of exclusive Strombolicchio haplotype compared with those found in Palinuro/Capri (i.e., two mutation steps). The presumed source population, likely distributed along the south Tyrrhenian coastline, could be extinct today or even still existing. Despite the long history of floristic investigations in the Mediterranean Basin, the existence of unknown populations of *E. saxicola* could not be excluded due to the difficulty in recognizing the species (Strumia et al., 2015). Implementation of botanical surveys along the South Tyrrhenian coastlines, considering the Sea surface circulation and suitable rocky habitats, could fill this gap. Furthermore, a previous study on the genus *Limonium* showed that rising sea levels during the warm phases of the glaciation had led to the steady decline of coastal populations (Koutroumpa et al., 2021). We, therefore, cannot exclude that this flooding may have played a similar role in the extinction of some *E. saxicola* populations along the coastline.

### What Determines the Genetic Diversity and Genetic Structure of Extant Populations?

The complete analysis of plastid genome variation revealed partial admixture and a very low level of between population differentiation (only 1–2 mutation steps). The different analyses of nuclear markers, performed with different settings, concordantly shows three main genetic clusters matching the three sampling localities of Palinuro, Capri, and Strombolicchio (Figure 2C). In contrast to plastidial admixture, the nuclear markers revealed a present story of strong genetic isolation for the three living populations (Figures 2–4; Table 3). Indeed, all sampled individuals have been correctly assigned to their source population in all analyses, thus almost indicating a complete absence of genetic admixture and ongoing gene flow (with corresponding high Fst values).

MIGRATE and BayesAss estimates revealed that historical (mh) and contemporary (mc) levels of migration between the three genetically distinct populations are very small and similar in magnitude (Supplementary Table 2), as also supported by the large generation times estimated by the simulation performed in DIYABC. The low levels of contemporary migration are not surprising because geographic distance and cliff habitat make dispersal between populations highly unlikely. However, as historical migration rates are also very low, concordant patterns strongly imply that the high levels of genetic structure currently observed among extant populations stem from a low colonization ability, which thus seems to be a long-standing life-history trait of *E. saxicola.* One evolutionary implication of similar low contemporary and historical migration rates over time is that *E. saxicola* has a long history of living in relatively small, isolated populations (still preserving enough genetic variation). In contrast to other island narrow endemics (Molins et al., 2009; Mayol et al., 2012; López-Pujol et al., 2013), for which the sea level oscillations represented a switch-off-switch-on the barrier to gene flow, sea levels theoretically do not represent a major barrier for seed dispersal of *E. saxicola*, as well as for other halophytes of both sandy and rocky coasts. Nonetheless, phases of sea lowering could have led to both the increase in available habitat through land emersion and the promotion of hopping colonization by the decrease in distance between isolated populations. However, phases of the current sea rising do not totally halt the potential dispersion of *E. saxicola* but decrease the probability of its occurrence due to the increased distance between a few suitable sites.

The DIYABC analysis identified two introductions temporally separated from the Palinuro to Capri, and from there to Strombolicchio (DIYABC model selection, Scenario 1) as the most likely hypothesis for *E. saxicola* presents distribution. By assuming a generation time of 10 years, we can consider that the introduction to Capri, approximately 0.25 Mya, and the colonization of Strombolicchio (0.09 Mya) took place around the late Pleistocene. The presence of inaccessible known plant spots (on Capri) and the possible occurrence of other unknown plant spots along the Palinuro cliffs suggest we probably have underestimated the census population sizes (N) for the three populations. Still, Ne estimations are near and even larger than N (Table 3), i.e., most of the individuals are breeding individuals in the population. Overall, the effective population size of *E. saxicola* is historically low because climatic oscillations during the quaternary glacial-interglacial cycles periodically forced populations to track their optimum into smaller cliff areas of suitable habitat. The almost exclusive preference of *E. saxicola* for north-facing cliffs suggests an adaptation to cooler conditions, which further highlights the possibility of different phases of population expansion/contraction during these oscillation cycles.

### Can the Combination of Phylogeography and Genetic Diversity Help Define *E. saxicola* Conservation Priorities?

While geographic isolation seems the obvious explanation for the current population genetic structure, it partly contrasts with the levels of genetic diversity still detected within each population. Such genetic diversity within populations is unlikely to have resulted from recent or contemporary gene flow but rather points at relative demographic stability (as suggested by Ne estimations) both in recent and ancient timescales. All sampled individuals, despite a clumped distribution due to the accessibility of the cliff faces, were genetically different, indicating that clonality in this species is rare. Moreover, at least at the intrapopulation level, the long lifespan of adult plants, together with their high resprouting ability and wind pollination, may contribute to maintaining a high level of outcrossing and multiple random mating among the few individuals (i.e., observed heterozygosity was comparable to the expected one). Interestingly, wind pollination is an almost unique trait among Mediterranean narrow endemics. Indeed, anemophily is a plesiomorphic trait in *E. saxicola* and Amarantaceae, whereas, in the few other known wind-pollinated endemics, this mechanism is rather a secondary adaptation to the low availability of biotic pollination (Traveset and Navarro, 2017).

Regardless of the extremely small number of individuals in each population, the moderate degree of intrapopulation co-ancestry (Figure 5) suggests that a consistent level of crosses among unrelated individuals occurs. Even if the reproductive success of individual plants is unknown, the pattern of genetic relatedness among individuals of these small populations suggests that most of the adult plants contribute to the next generation, i.e., that random mating occurs. This reproductive behavior is consistent with the observation of Ho not different from He and a moderate excess of homozygotes in all populations compared with Hardy–Weinberg equilibrium expectations (Fis average, 0.10). The relative excess of homozygotes (higher Fis) observed in Palinuro denotes some heterogeneity in this mainland population compared to the islets, consisting of populations composed of several subgroups (the so-called Wahlund effect). Indeed, in contrast to the other two insular populations sampled on single cliffs, the Palinuro population was sampled along different cliffs. This subpopulation structure is also partially evident in the heatmap graph of Fst (Figure 4).

As reported for other extremely narrow endemics (see Médail and Baumel, 2018), *E. saxicola* still maintains some degree of genetic diversity even at the intrapopulation level (despite its small population sizes); this genetic trait may have represented life insurance for the long-term survivorship of this rare species. This survivorship was further aided by the low level of anthropogenic disturbance and high stability typical of cliff plant communities (Davis, 1951). Indeed, maintaining genetic diversity can reduce the probability of extinction of small populations by providing the standing genetic variation for local adaptation (Leimu and Fischer, 2008). Therefore, the present Palinuro population still holds enough genetic diversity to represent a valuable “donor population” for future reintroduction programs of this species. The high heterozygosity can be relevant in terms of conservation guidelines as genetic diversity of *E. saxicola* can be preserved in the long term even in its current isolated populations, confirming that narrow endemics are not necessarily “evolutionary dead-ends,” but rather may represent species that have a strong evolutionary legacy (Médail and Baumel, 2018). From a conservation perspective, we suggest that, at least in the short term, genetic factors may have little impact on the persistence of small populations of this rare paleoendemic species, but, rather, ecological factors (as persistence of suitable habitat) may play a larger role in determining whether populations survive in the long term. In this respect, both the IUCN category and the potential threats of extinction of this species (Santangelo et al., 2012; Orsenigo et al., 2018) should be reconsidered.

## Conclusion

Even if short and long seed dispersion is still possible, why *E. saxicola* is so rare compared with other littoral halophytes species (Davis, 1951)? Indeed, the strong genetic differentiation detected among living populations suggests that the long-distance colonization typical of halophytes is merely occasional. We recognize that, independently of its colonization potential (through hydrochory), the rarity of this species must be considered also in terms of its specific requirements for habitat type as well as its biological features. Currently, *E. saxicola* has distributed exclusively on north facing (± 45° deviation from North) rocky shores (Strumia et al., 2015), but we do not have experimental evidence to support this restricted distribution. Since long-distance dispersal and colonization are different processes [see Feliner (2014) and references therein], we hypothesize that other factors determine the successful long-distance colonization in *E. saxicola*: namely, landing in a suitable microhabitat, seed germination, and, finally, seedling anchorage (Strumia et al., 2020b). Thus, the rarity of *E. saxicola* must be attributed not only in terms of the longevity and the ability of seeds to float but also, if not predominantly, of the habitat suitability of reached cliffs. These findings confirm the distinctiveness of *E. saxicola* from other halophytes and narrow endemics of the Mediterranean Basin. In conclusion, *E. saxicola* shows habitat specificity with individuals found predominately in small and sporadic populations. However, anemophilous pollination maintains a high-genetic diversity in this species, thus preventing the rapid decline, which is usually expected in small populations, thus ruling out the possibility of genetic impoverishment and inbreeding depression as immediate causes of threat.

## Data Availability Statement

The datasets presented in this study can be found in online repositories. The names of the repository/repositories and accession number(s) can be found below: BioProject PRJNA756897.

## Author Contributions

SS, AS, and DC: conceptualization. SS and AS: plant material collection. SC and DC: data curation. SC, DC, and TG: formal analysis. DC and SS: funding acquisition. DC and TG: methodology. SS, SC, DC, and AS: writing – original draft preparation. All authors have read and agreed to the published version of the manuscript.

## Conflict of Interest

The authors declare that the research was conducted in the absence of any commercial or financial relationships that could be construed as a potential conflict of interest.

## Publisher’s Note

All claims expressed in this article are solely those of the authors and do not necessarily represent those of their affiliated organizations, or those of the publisher, the editors and the reviewers. Any product that may be evaluated in this article, or claim that may be made by its manufacturer, is not guaranteed or endorsed by the publisher.

## Footnotes

1
www.bioinformatics.babraham.ac.uk/projects/fastqc

2
https://github.com/enormandeau/Scripts/blob/master/fastqCombinePairedEnd.py

3
http://tree.bio.ed.ac.uk/software/figtree
