# Supplementary material for: Hopping or Jumping on the Cliffs: The Unusual Phylogeographical and Demographic Structure of an Extremely Narrow Endemic Mediterranean Plant
Source: Front Plant Sci. 2021 Nov 10;12:737111. doi: 10.3389/fpls.2021.737111 (PMC8631297; doi:10.3389/fpls.2021.737111)
Supplement: Supplementary file 1 [file Data_Sheet_1.pdf]

Supplementary Material

1 Supplementary Data

1.1 Effects of SNPs filtering strategies

Since an intrinsic source of bias is generally associated with library prep (i.e. low DNA quality and batch effect) sequenced samples differed in the amount of generated reads and coverage (**Table SM1).** This discrepancy is generally overcome by applying a different filtering set to the samples and called SNPs. It is well known that different filtering strategies influence both the estimation of genetic diversity and differentiation (see Cozzolino et al. 2020, Gargiulo et al. 2021). Therefore, the testing of robustness and reliability of results of ML tree, pairwise Fst and co-ancestry among individuals and populations, were also calculated with a reduced dataset (25 individuals, by selecting 120 SNPs shared by at least 95% of accessions) (**Supplementary Figures 1, 2** and **3**).

We also estimated how global F_{ST} and overall EC (estimated co-ancestry) vary depending on the number of SNPs (and missing data) by analyzing matrices with SNPs present in at least 10%, 30%, 70%, 90% and 95% of individuals. Less stringent filtering (e.g. loci shared by 10-30% of individuals) preferentially retains more population-specific loci (loci with high mutation rate/substitution rate). These latter loci, with a higher amount of missing data, are therefore those that have differentiated among diverged populations (so generating a higher F_{ST}). Instead, when loci with missing data are excluded in favor of more highly represented, and thus more conserved loci, (loci shared by 95% of individuals), these latter are shared among diverged populations and reduce the overall F_{ST} value (see Cozzolino et al., 2020). For similar reasons, increasing the number of SNPs (i.e. loci shared by 10-30% of individuals) determined an increase in the overall EC values for several pairs of individuals (Gargiulo et al., 2021) (**Supplementary Figure 4 A** and **B**).

Supplementary Material

Table SM1. Details of *Eokochia saxicola* ddRAD and plastid (cpDNA) datasets.

population	ID sample	ddRAD dataset with 48 samples	ddRAD dataset with 25 samples	n. raw reads of ddRAD sample	n. mapped reads of ddRAD sample	Plastidial dataset	n. raw reads of cpDNA sample	n. mapped reads of cpDNA
Strombolicchio	K1-1	X		152363	77352			
Strombolicchio	K1-2	X	x	601763	266724	x	2052524	159632
Strombolicchio	K1-3	X		343219	127567			
Strombolicchio	K1-4	X		176813	57916			
Strombolicchio	K2-1	X	X	707682	255225			
Strombolicchio	K2-2	X	X	1962415	169623			
Strombolicchio	K3-1	X	X	709119	275066			
Strombolicchio	K3-2	X	X	735656	293955	x	1197670	111080
Strombolicchio	K4-1	X		187324	65300			
Strombolicchio	K5-1	X	X	912034	435721			
Strombolicchio	K5-2	X	X	598354	224217	x	1696902	167310
Strombolicchio	K5-3	X		122340	67951			
Capri	C1.1	X		190668	69853			
Capri	C1.2	X	X	927867	361201	x	1104447	148554
Capri	C1.3	X	X	1531318	246937			
Capri	C1.4	X	X	573096	140196			
Capri	C1.5	X		381389	173661	x	6456625	1032602
Capri	C1.6	X	X	866657	266134			
Capri	C1.7	X	X	877895	384465			
Capri	C1.8	X		207483	85042			
Capri	C1.9	X	X	654698	295385			
Capri	C1.10	-	-	-	-	x	1543864	155254
Capri	C1.11	X		169009	62016			
Capri	C1.12	X		60436	25263	x	1243438	121250
Capri	C1.13	X		62267	26719			
Capri	C1.14	X		357662	186595			
Capri	C1.15	X		377649	155077			
Capri	C1.16	X	X	673012	281211	x	3437057	541486
Capri	C1.17	-	-	-	-	x	3374138	388062
Capri	C1.18	X	X	1690863	91207			
Palinuro	CAM1	X		413603	272974			
Palinuro	CAM2	X		52598	29237			
Palinuro	P1	X		215954	94888	x	1540532	201192
Palinuro	P2	X	X	1416236	571689			
Palinuro	P3	X	X	863162	381993	x	3806113	606896
Palinuro	P4	X	X	732001	354902			

Palinuro	P5	X		112659	58447			
Palinuro	P6	-	-	-	-	x	727584	84046
Palinuro	P7	X	X	840168	369839			
Palinuro	P8	X	X	820162	383342			
Palinuro	P9	X		50003	24111			
Palinuro	P10	X	X	584174	256058			
Palinuro	P11	X		296303	131427			
Palinuro	P12	X		216499	100217			
Palinuro	P13	X	X	1133910	514416			
Palinuro	P15	X	X	1254475	545498			
Palinuro	P16	X	X	1363914	617533	x	1801655	95508
Palinuro	P17	X		304916	143022			
Palinuro	CFK5	X		99917	43198	x	1480751	161854
Palinuro	CFK6	X		70578	21094	x	5103918	639474
Palinuro	PIK1	X	X	1623784	701128	x	4240074	544078

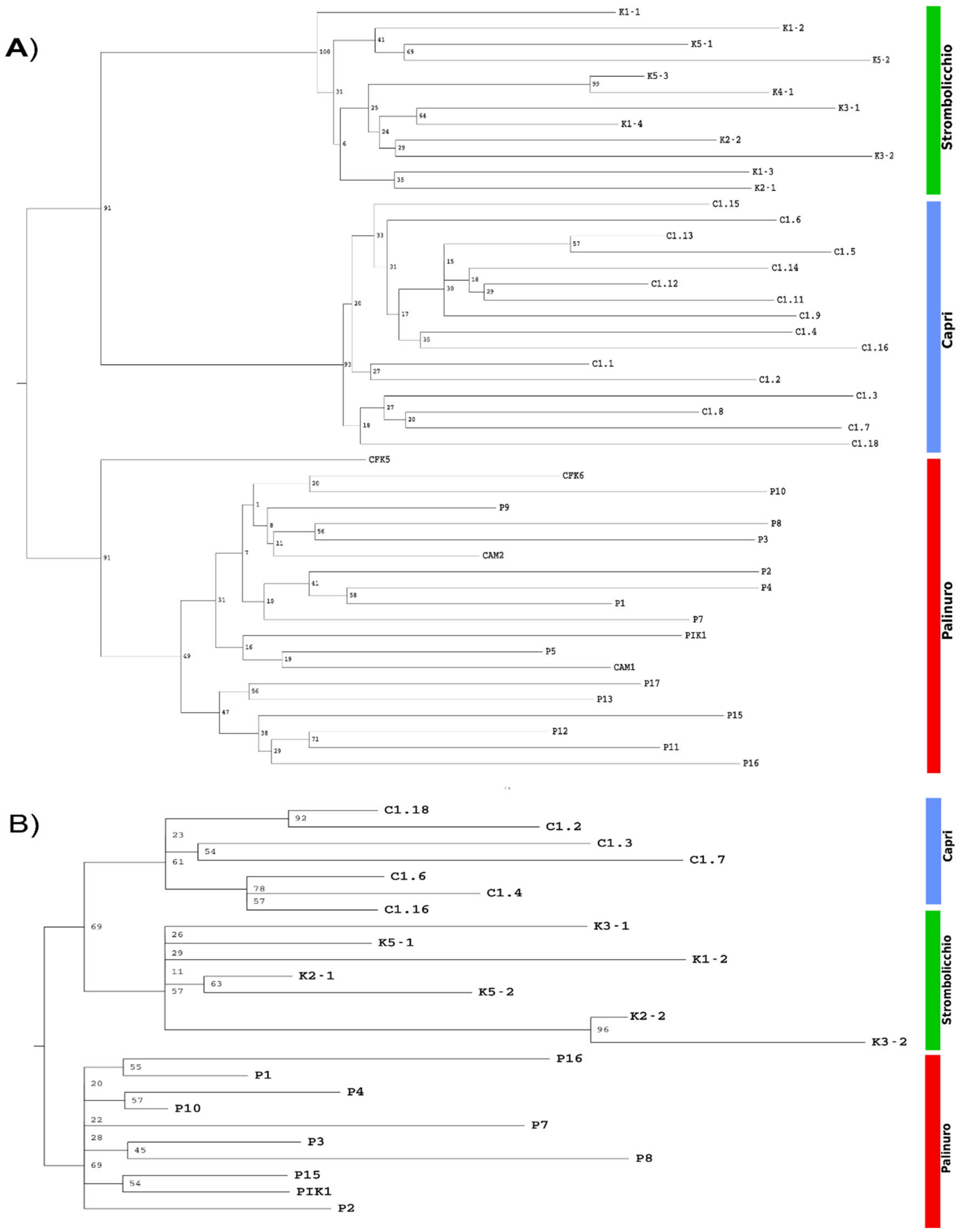

Supplementary Figure 1. A) Phylogenetic tree using 48 individuals (SNPs present in at least 70% individuals). B) Phylogenetic tree using 25 individuals (SNPs present in at least 95% individuals). Numbers associated with branches are ML bootstrap supporting values.

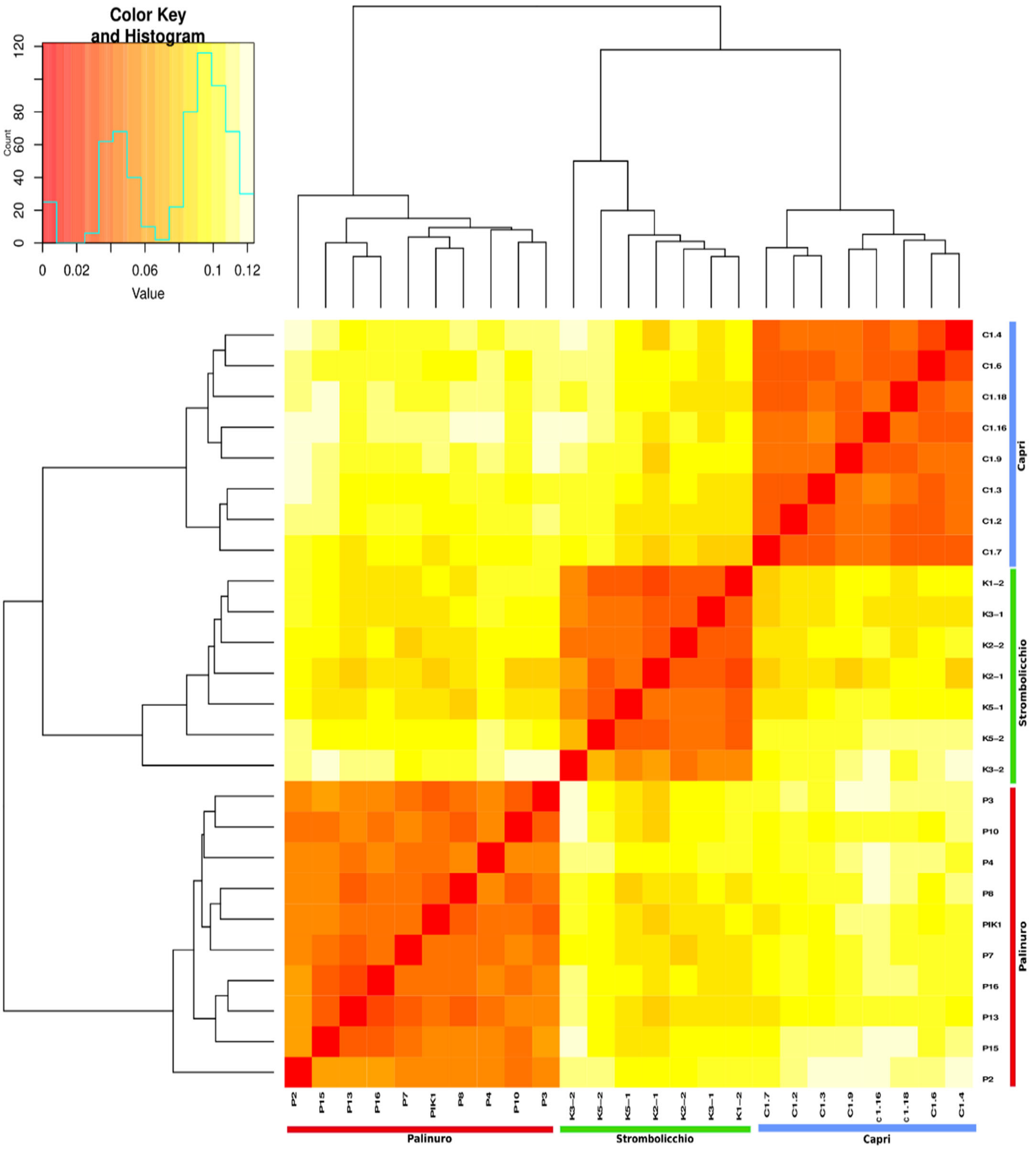

Supplementary Figure 2. Heatmap graph of Fst built by using 120 SNPs across 25 accessions (SNPs present in at least 95% individuals).

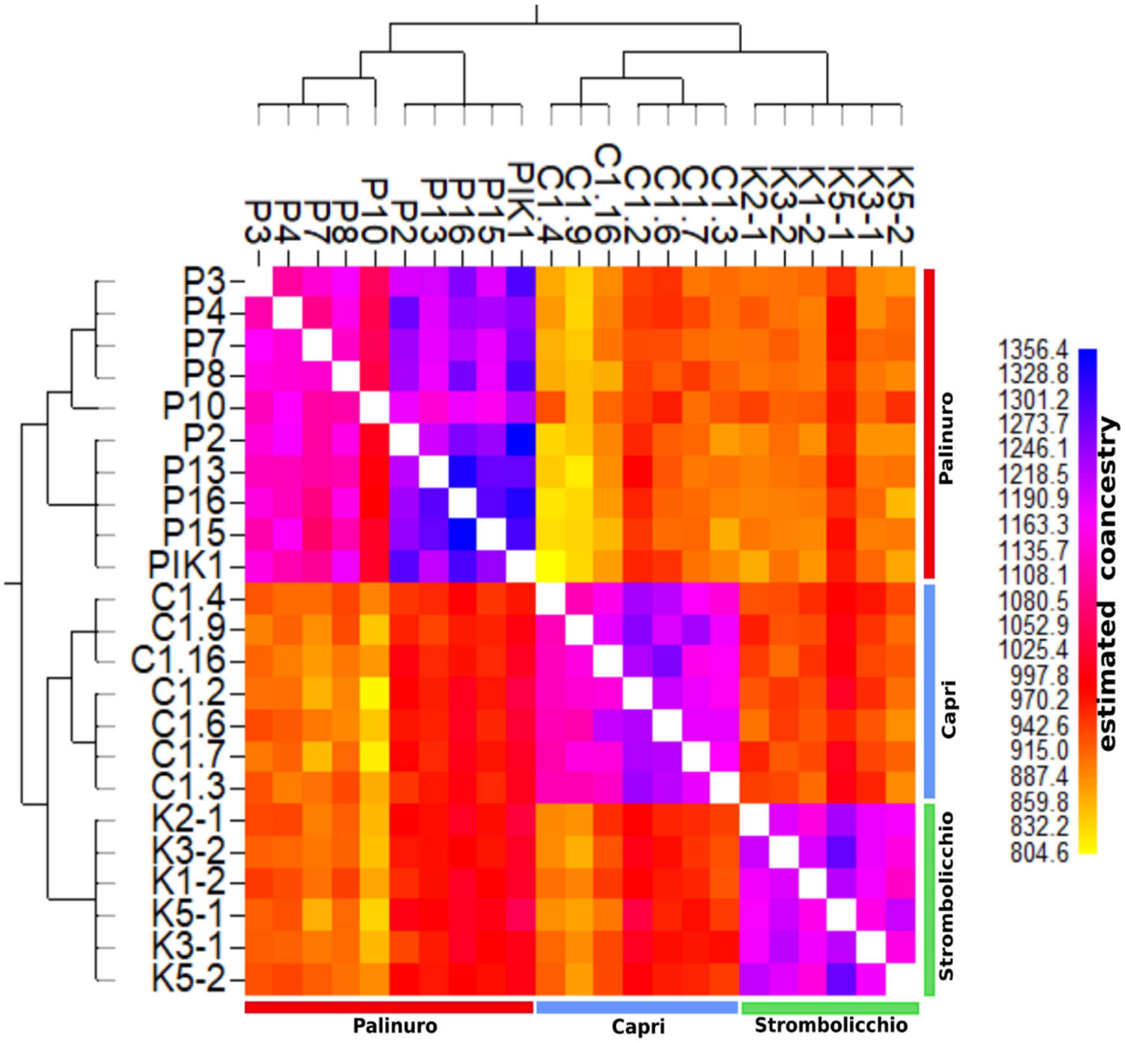

Supplementary Figure 3. Co-ancestry matrix shared among 25 accessions (SNPs present in at least 95% individuals).

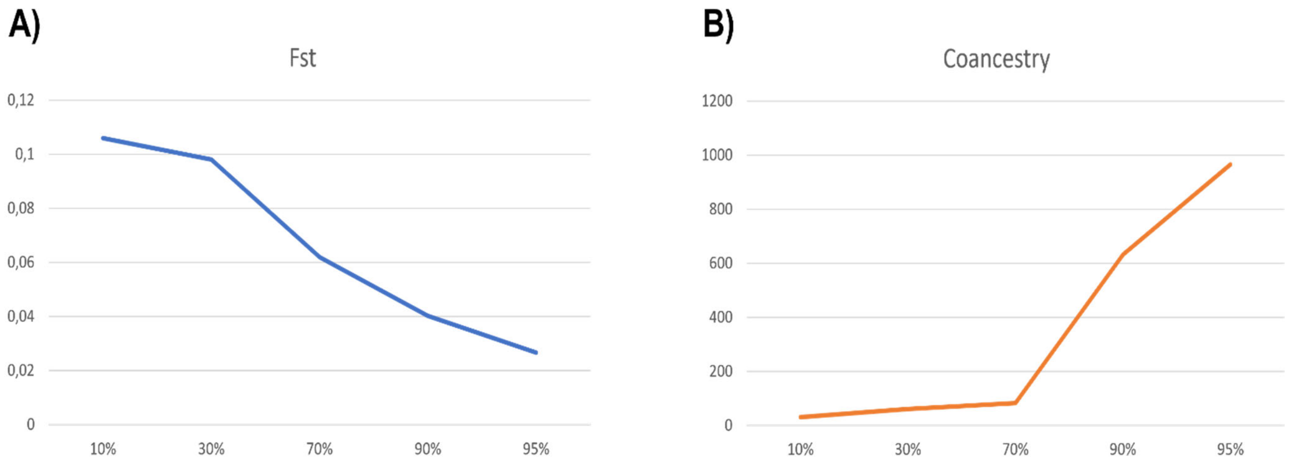

Supplementary Figure 4. A) Global Fst values among the three *Eokochia saxicola* populations by using matrices with SNPs present in at least 10%, 30%, 70%, 90% and 95% of individuals. B) Average co-ancestry values in fineRADStructure matrices with SNPs present in at least 10%, 30%, 70%, 90% and 95% of individuals).

1.2 Discriminant Analysis of Principal Components (DAPC)

The Discriminant Analysis of Principal Components (DAPC) was implemented using the R package adegenet v2.02 (Jombart, 2008). The dataset of 48 samples were first transformed through a PCA and then the discriminant analysis (DA) was performed on the retained principal components (PCs). The number of retained PCs were chosen from two approaches, the a-score optimization and cross-validation, implemented with functions optim.a.score and xvalDapc respectively (**Supplementary Figure 5 A** and **B)**. Based on the model validation, the 'optimum' n. PCs in the DAPC analysis associated with the lowest RMSE (0.043) was 20. The clusters were subsequently identified with the find.clusters function. The best K was determined using the Bayesian Information Criterion (BIC) approach (**Supplementary Figure 6)**. From the clustering result, the memberships probability of each individual to the clusters were plotted in R implementing the compoplot function (**Supplementary Figure 7).**

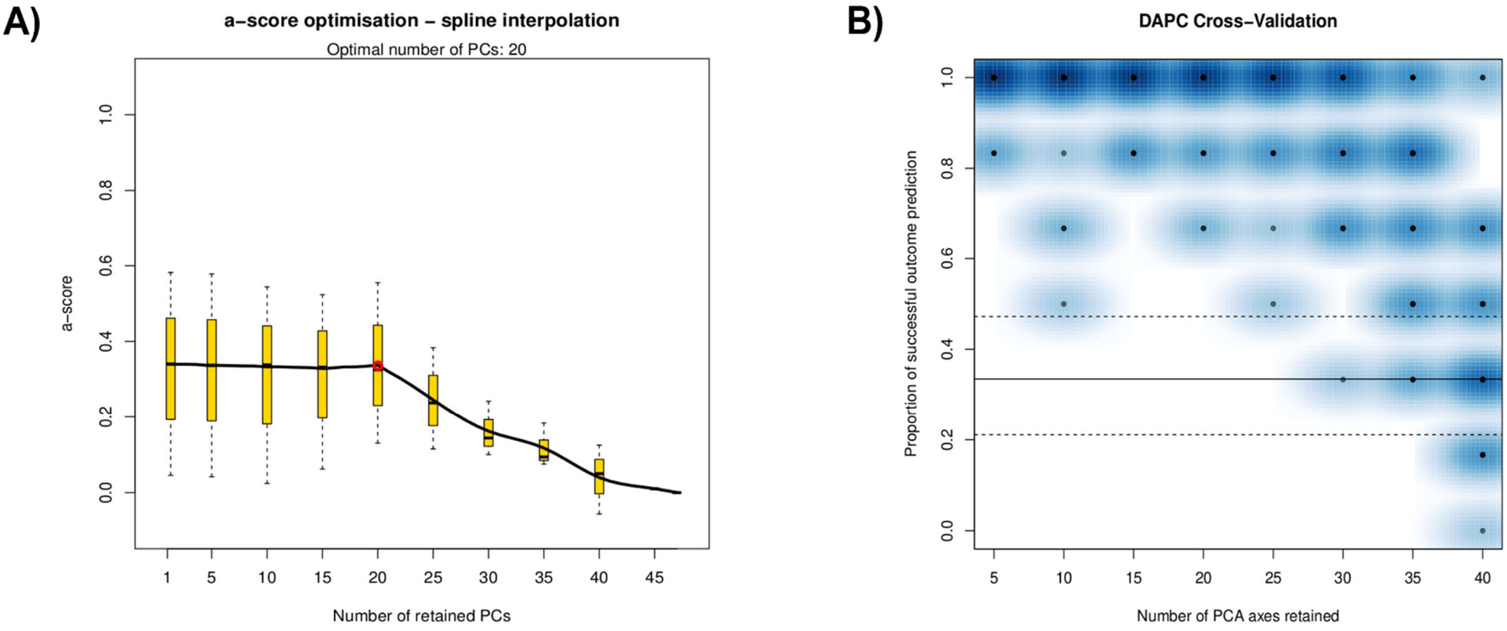

Supplementary Figure 5. A) a-score optimization and B) the cross-validation procedure aimed to identify the number of PCA components that should be retained for the DAPC (i.e. the number of components that maximize successful individual assignation to the k clusters).

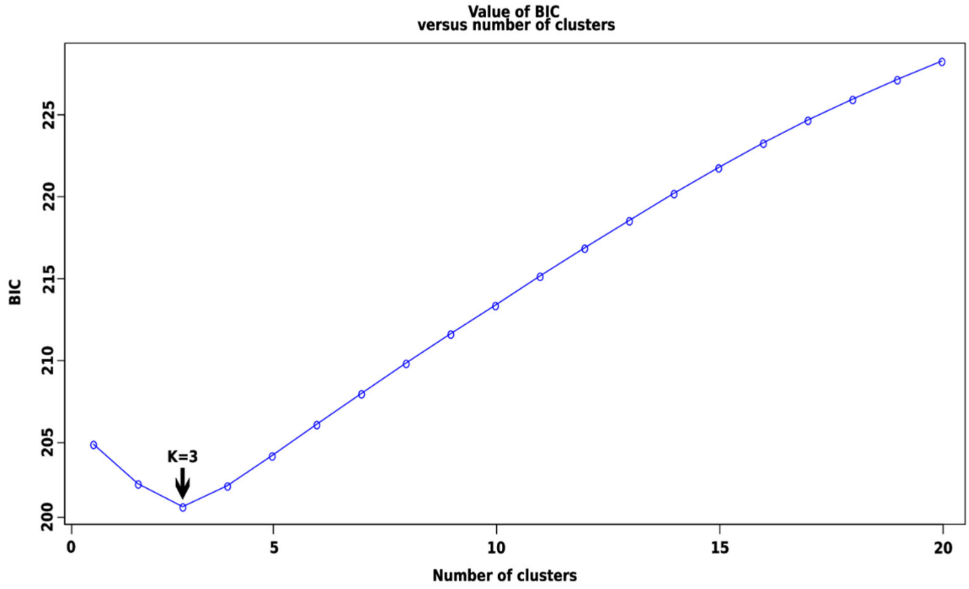

Supplementary Figure 6. Discriminate analysis of principal components (DAPC). The analysis was drawn using 3962 SNPs (Single Nucleotide Polymorphism) across 48 accessions and was constructed using 20 principal components (PCs) and two discriminate functions. The scree plot of eigenvalues

(inset) indicates eigenvalues of discriminant analysis and the amount of variation contained in the different principal components. The lower BIC value corresponded to the best K=3 (number of cluster).

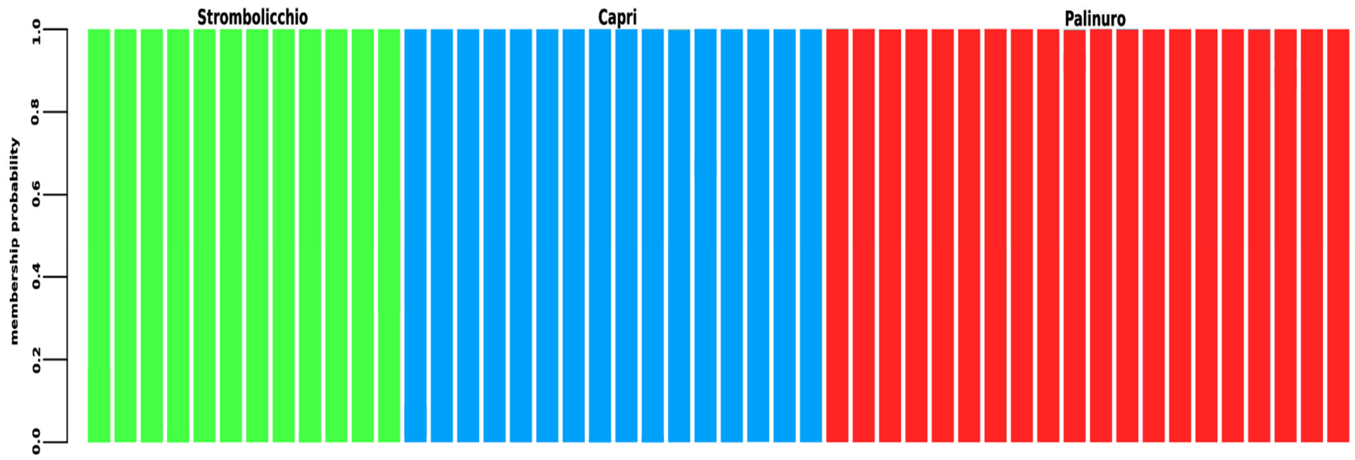

Supplementary Figure 7. A barplot depicting the probabilities of assignment of individuals to K=3 genetic DAPC clusters. Each bar corresponds to an individual, with colors denoting sampling origin.

1.3 Contemporary migration rates

The contemporary migration rates were estimated from the current generation and two past generations using Bayesian inference in BayesAss v3 (Mussmann et al., 2019). Preliminary runs were performed to adjust the mixing parameters, starting from a different allele frequency (set at 0.05, 0.10, 0.25 and 0.50) and migration rate (set at 0.001, 0.01 and 0.1) and with 0.10 of inbreeding coefficients (as mean value of Fis estimated from gene diversity). Following authors' recommendations, we selected the run which would ensure proposal acceptance rates of mixing parameters between 20% and 60% (**Supplementary Figure 8**).

Furthermore, we ran a MCMC with 10 million interactions, discarding the first three million iterations and sampling every 1000 iterations from the remaining nine million. In total, we generated 9000 observations from the chain that was used to estimate our parameters (as indicated in https://github.com/brannala/BA3/blob/master/doc/BA3Manual.pdf). We performed this analysis from six independent runs with different seeds (default, 12345, 3468, 0125, 2341, 4321). Convergence of runs were examined by comparing the traces of each run using Tracer v1.6 (Rambaut et al., 2018) and by evaluating the Effective Sample Sizes (ESSs) of each parameter, keeping only runs where ESS≥200 (Nylander et al., 2008) (**Table SM2** and **Supplementary Figure 9**).

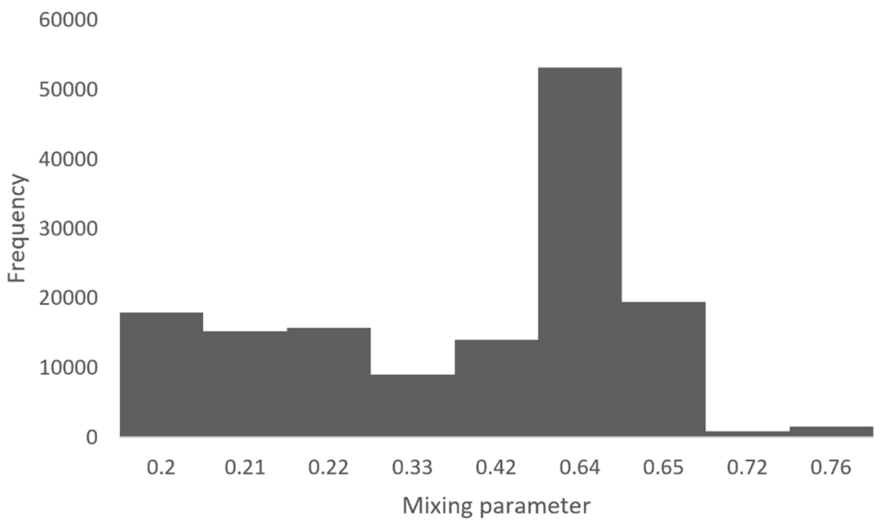

Supplementary Figure 8. The histogram plot showing the optimal mixing parameters for allowed acceptable thresholds as described in BayesAss manual.

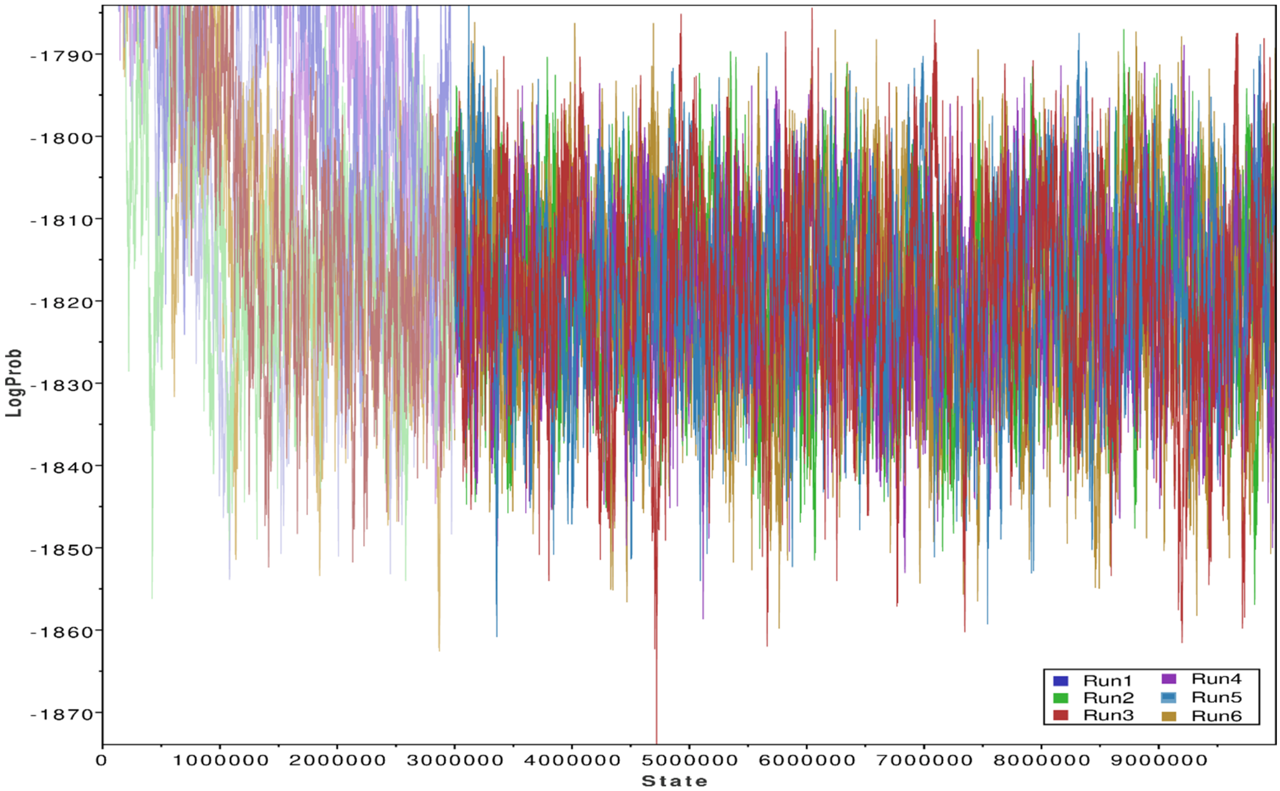

Supplementary Figure 9. Trace plot for Log probability of six runs created with the Tracer software.

Table SM2. Mean (Lower and Upper 95% CI, Confidence Interval) and Effective Sample Sizes (ESS) of recent migration rate of six runs estimated among 25 accessions with BayesAssv3. Abbreviation: CI, confidence interval; ESS, Effective Sample Sizes; P, Palinuro; K, Strombolicchio; C, Capri.

		RUN1				RUN2				RUN3			
		Mean	Lower 95% CI	Upper 95% CI	ESS	Mean	Lower 95% CI	Upper 95% CI	ESS	Mean	Lower 95% CI	Upper 95% CI	ESS
Migration into P	from C	0.0341	1.38E-05	0.0954	964	0.0330	6.25E-07	0.0910	1066	0.0334	6.81E-06	0.0952	905
	from K	0.0263	1.83E-02	0.0330	918	0.0255	1.55E-02	0.0332	842	0.2658	1.87E-02	0.0329	860
Migration into C	from P	0.0258	5.13E-06	0.0736	1007	0.0256	1.34E-06	0.07	2127	0.0250	3.96E-07	0.0716	2443
	from K	0.0630	1.83E-02	0.1303	935	0.0545	1.55E-02	0.3316	820	0.0658	1.87E-02	0.3298	950
Migration into K	from P	0.0341	1.38E-05	0.0954	1009	0.0330	6.25E-07	0.0910	905	0.0334	6.81E-06	0.0952	1063
	from C	0.0520	6.31E-07	0.0724	2219	0.0470	8.76E-06	0.0703	2512	0.0259	3.33E-06	0.0754	2300

		RUN4				RUN5				RUN6			
		Mean	Lower 95% CI	Upper 95% CI	ESS	Mean	Lower 95% CI	Upper 95% CI	ESS	Mean	Lower 95% CI	Upper 95% CI	ESS
Migration into P	from C	0.03160	1.56E-06	0.09090	967	0.03180	3.58E-06	0.08920	724	0.03300	3.25E-06	0.09390	952
	from K	0.02686	1.93E-02	0.033	826	0.02562	1.64E-02	0.03297	545	0.02668	1.86E-02	0.03307	912
Migration into C	from P	0.0263	3.94E-06	0.08	2190	0.0257	8.95E-07	0.07	2191	0.0257	2.90E-06	0.07	2395
	from K	0.06860	1.93E-02	0.33000	971	0.05620	6.43E-02	0.3297	552	0.06680	1.86E-02	0.33070	804
Migration into K	from P	0.03160	1.56E-06	0.09090	976	0.03180	3.58E-06	0.09	1149	0.03300	3.25E-06	0.09390	943
	from C	0.02620	5.02E-07	0.07540	2246	0.02530	6.80E-07	0.07	2345	0.02570	1.65E-06	0.07330	2222

1.4 Historical migration rates

Historical migration rate was estimated using Bayesian inference in MIGRATE (Beerli, 2006). Model parameters were set in Migrate-n using the Equal Migration model, whereas all populations had the same directional effect on gene migration. The geographic distance matrix file was imported into MIGRATE to scale migration rate parameter estimates using geographic distance. We assumed a Brownian motion model with constant mutation rates for all loci and we set to 10 long chains with 100,000 interactions, sampling every 100 steps for each locus and 10,000 discarded trees per chain since our populations sizes are relatively small (Beerli, 2015; Samarasin et al., 2017). Moreover, we ran two parallel runs with four heating chains (static, four parallel chains), with independent random starting points.

A uniform distribution prior with a range of 0–500 was then used for estimating immigration parameter M among populations, and a uniform distribution prior with a range of 0-0.100 was used for estimating θ (=4Neμ) within populations. Posterior distributions were generated using the Metropolis-Hasting algorithm. Convergence on stationary distributions of parameters was assessed based on the similarity of posterior distributions of the two independent runs, and the effective sample size (ESS) (**Table SM3)**. Finally, the historical rates of migrants per generation was estimated as $N_e m = \theta M/4$ (in MS supplementary Table 2).

Table SM3. Mean (Lower and Upper 95% CI, Confidence Interval) and Effective Sample Sizes (ESS) of historical immigrant (M) and θ of two runs and the combined run, estimated from MIGRATE among 25 accessions. Abbreviation: CI, confidence interval; ESS, Effective Sample Sizes; P, Palinuro; K, Strombolicchio; C, Capri.

	RUN 1				RUN 2				Combined			
Param-eter	Mean	Lower 95% CI	Upper 95% CI	ESS	Mean	Lower 95% CI	Upper 95% CI	ESS	Mean	Lower 95% CI	Upper 95% CI	ESS
θ K	0.00003	0	0.00007	2123758.08	0.00003	0	0.00007	2443526.00	0.00003	0	0.00007	2403410.42
θ C	0.00003	0	0.00007	2004127.28	0.00003	0	0.00007	2178382.00	0.00003	0	0.00007	2164485.96
θ P	0.00005	0	0.00013	2066539.01	0.00005	0	0.00013	1823850.00	0.00005	0	0.00013	1974095.01
M C-K	471.1	459.3	478.0	16669117.17	468.8	459.0	478.0	16112013.00	468.9	459.3	478.0	17030718.24
M P->K	71.8	60.7	78.0	15531701.61	68.8	60.7	78.0	16295566.61	70.1	60.7	78.0	16867837.32
M K->Ci	6.1	4.1	8.0	16956451.07	5.5	4.0	7.0	17771842.16	5.3	4.7	8.0	17395318.19
M P->C	461.2	458.7	478.0	15323821.32	468.4	458.7	478.0	17203202.73	471.2	458.7	478.0	16927393.56
M K->P	4.5	3.3	6.0	17086739.02	5.9	3.7	8.0	15446772.68	4.6	4.3	9.0	17532427.34
M C->P	46.6	45.1	50.0	17194566.50	48.2	46.4	50.0	18116924.23	46.2	45.3	47.0	17231776.80

1.5 Reconstruction of demographic historical scenarios

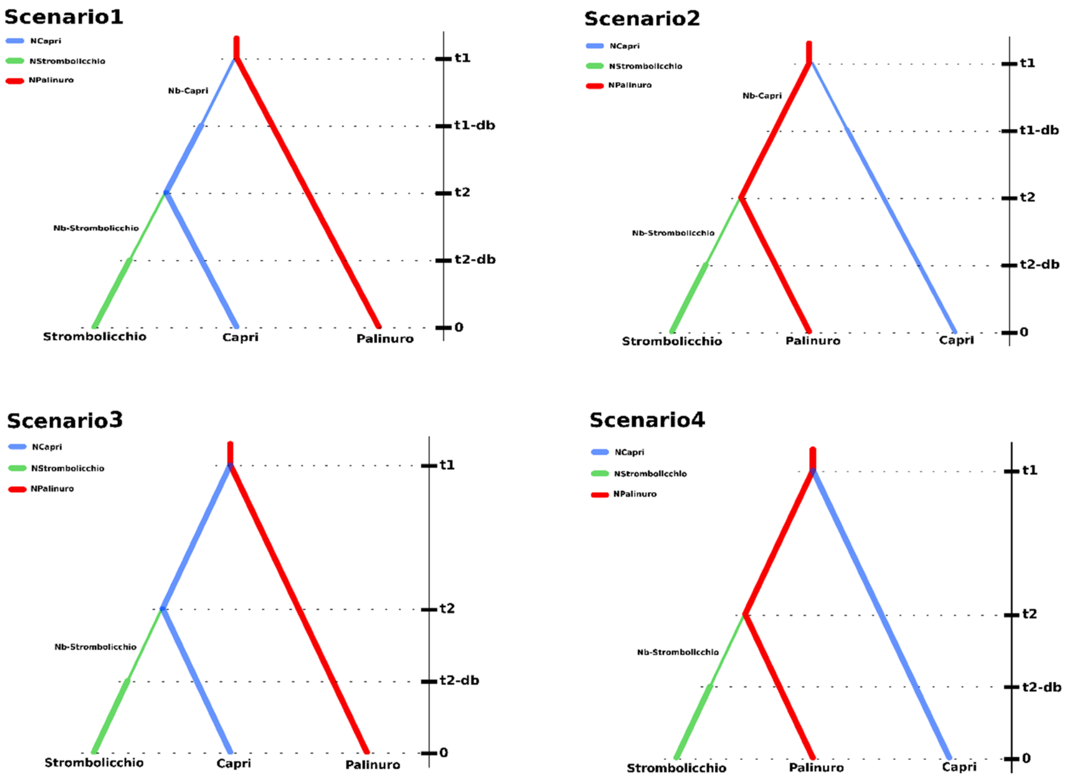

Supplementary Figure 10. Demographic history of *Eokochia saxicola* populations implemented by DIY ABC. The branch colors indicate discrete population size parameters in the model. (Nb): number of founders (from 10 to 50 individuals); (t1 and t2): time of the split event (from 10 to 50,000 generations); (t-db): bottleneck time after the split event (from 10 to 100 generations). Note: time is measured in generations and is not to scale

Scenario 1: t1 represents the split between Palinuro and Capri while t2 represents the split between Capri and Strombolicchio. The thin branch width indicates bottlenecks of duration db (t1-db and t2-db) with effective population sizes of Nb (Nb-Capri and Nb-Strombolicchio).

Scenario 2: t1 represents the split between Capri and Palinuro while t2 represents the split between Palinuro and Strombolicchio. The thin branch width indicates bottlenecks of duration db (t1-db and t2-db) with effective population sizes of Nb (Nb-Capri and Nb-Strombolicchio).

Scenario 3: An ancestral population split in Capri and Palinuro populations at time t1 while t2 represents the split between Capri and Strombolicchio. The thin branch width indicates a bottleneck of duration db (t2-db) with effective population sizes of Nb-Strombolicchio.

Scenario 4: An ancestral population split in Capri and Palinuro populations at time t1 while t2 represents the split between Palinuro and Strombolicchio. The thin branch width indicates a bottleneck of duration db (t2-db) with effective population sizes of Nb-Strombolicchio.

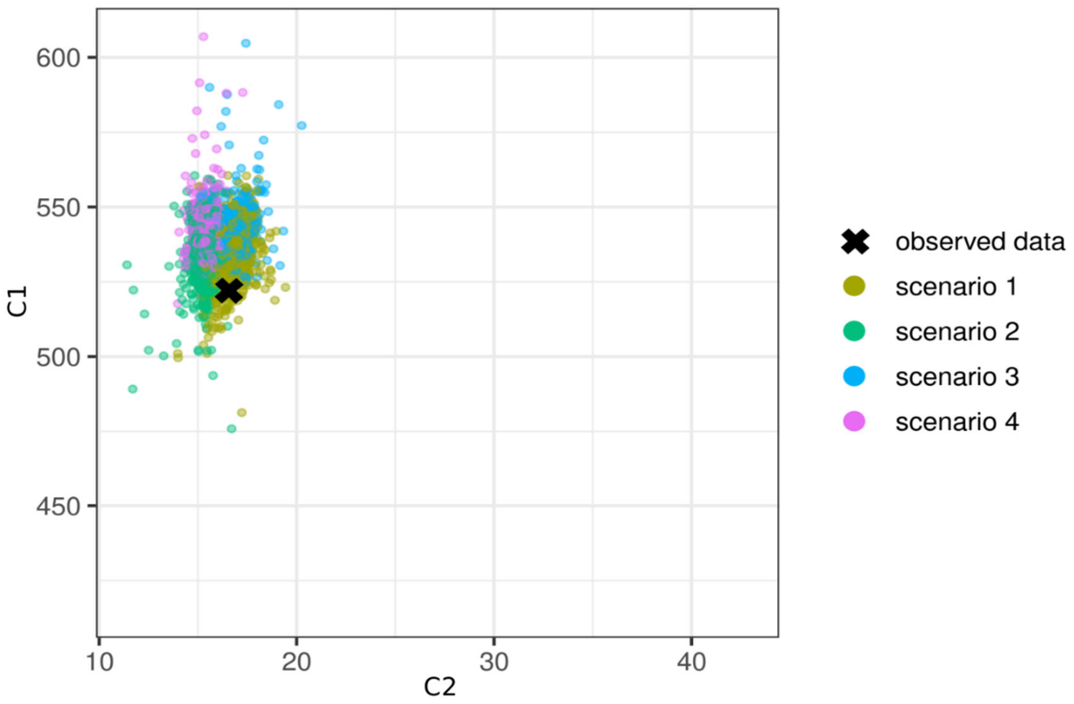

Supplementary Figure 11. Projection on the first two LDA axes of the observed dataset and the simulated datasets. Colors correspond to the group of scenarios. The location of the observed dataset (black star) suggests an association with the scenario 1.

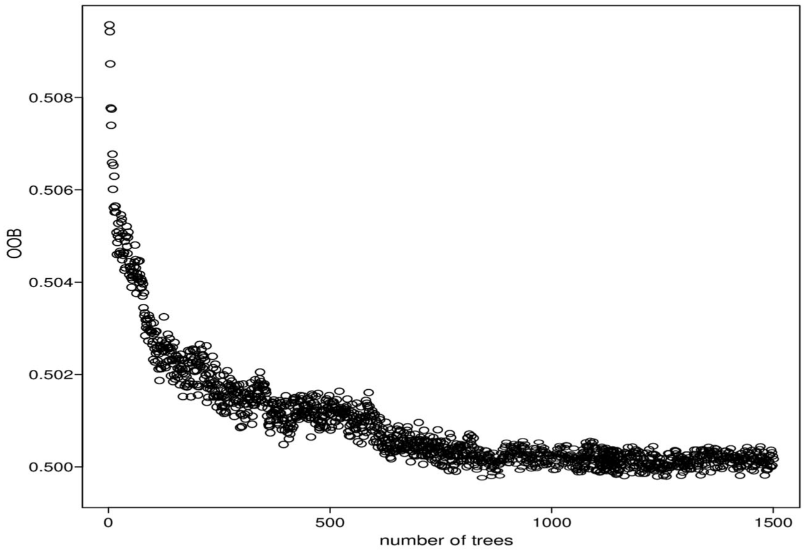

Supplementary Figure 12. Effect of the number of RF-trees for scenario choice. The effect of the number of trees in the forest on the prior error rate when comparing the four scenarios separately. The number of datasets simulated using DIYABC was 1,000,000. The shape of the curve shows that the prior error rate stabilizes for several RF-trees > 1,000.

2 Reference

Beerli, P. (2006). Comparison of Bayesian and maximum-likelihood inference of population genetic parameters. Bioinformatics 22, 341–345. doi:10.1093/bioinformatics/bti803.

Beerli, P. (2015). How to use MIGRATE or why are Markov chain monte Carlo programs difficult to use?. doi:10.1017/CBO9780511626920.004.

Collin, F. D., Durif, G., Raynal, L., Lombaert, E., Gautier, M., Vitalis, R., et al. (2021). Extending approximate Bayesian computation with supervised machine learning to infer demographic history from genetic polymorphisms using DIYABC Random Forest. Mol. Ecol. Resour. 1–16. doi:10.1111/1755-0998.13413.

Cozzolino, S., Scopece, G., Roma, L., and Schlüter, P. M. (2020). Different filtering strategies of genotyping-by-sequencing data provide complementary resolutions of species boundaries and relationships in a clade of sexually deceptive orchids. J. Syst. Evol. 58, 133–144. doi:10.1111/jse.12493.

Gargiulo, R., Kull, T., and Fay, M. F. (2021). Effective double-digest RAD sequencing and genotyping despite large genome size. Mol. Ecol. Resour. 21, 1037–1055. doi:10.1111/1755-0998.13314.

Jombart, T. (2008). Adegenet: A R package for the multivariate analysis of genetic markers. Bioinformatics 24, 1403–1405. doi:10.1093/bioinformatics/btn129.

Mussmann, S. M., Douglas, M. R., Chafin, T. K., and Douglas, M. E. (2019). BA3-SNPs: Contemporary migration reconfigured in BayesAss for next-generation sequence data. Methods Ecol. Evol. 10, 1808–1813. doi:10.1111/2041-210X.13252.

Nylander, J. A. A., Wilgenbusch, J. C., Warren, D. L., and Swofford, D. L. (2008). AWTY (are we there yet?): A system for graphical exploration of MCMC convergence in Bayesian phylogenetics. Bioinformatics 24, 581–583. doi:10.1093/bioinformatics/btm388.

Rambaut, A., Drummond, A. J., Xie, D., Baele, G., and Suchard, M. A. (2018). Posterior summarization in Bayesian phylogenetics using Tracer 1.7. Syst. Biol. 67, 901–904. doi:10.1093/sysbio/syy032.

Samarasin, P., Shuter, B. J., Wright, S. I., and Rodd, F. H. (2017). The problem of estimating recent genetic connectivity in a changing world. Conserv. Biol. 31, 126–135. doi:10.1111/cobi.12765.
