# Supplementary material for: Hopping or Jumping on the Cliffs: The Unusual Phylogeographical and Demographic Structure of an Extremely Narrow Endemic Mediterranean Plant
Source: Front Plant Sci. 2021 Nov 10;12:737111. doi: 10.3389/fpls.2021.737111 (PMC8631297; doi:10.3389/fpls.2021.737111)
Supplement: Supplementary file 2 [file Table_1.doc]

**Supplementary table 1**

| **Position​** | **REF​** | **ALT​** | **Location​** | **Samples​** |
| --- | --- | --- | --- | --- |
| 59799 | A​ | T​ | petA-psbJ​ | C1-2, K5-2, C1-10, CFK-5, C1-12, P1, P6, P16, C1-5, C1-16, C1-17, CFK6, P1-K1, P3​ |
| 76648 | G​ | C​ | intron rpl16​ | K1-2, K3-2​ |
| 1215451 | T​ | A​ | trnR-ACG​ | C1-10, CFK-5​ |

**Supplementary Tab. 1**: Annotation of identified mutations in plastid sequences. The table shows the position, the alternative base, the reference base, and individuals with corresponding mutation.
