# Supplementary material for: Hopping or Jumping on the Cliffs: The Unusual Phylogeographical and Demographic Structure of an Extremely Narrow Endemic Mediterranean Plant
Source: Front Plant Sci. 2021 Nov 10;12:737111. doi: 10.3389/fpls.2021.737111 (PMC8631297; doi:10.3389/fpls.2021.737111)
Supplement: Supplementary file 3 [file Table_2.doc]

**Supplementary table 2**

|  | | **Contemporary migration rate**  **(mc)** | | | **Historical migration rate**  **(mh)** | | |
| --- | --- | --- | --- | --- | --- | --- | --- |
|  |  | **Mean** | **Lower 95% CI** | **Upper 95% CI** | **Mean** | **Lower 95% CI** | **Upper 95% CI** |
| **Migration into Palinuro** | **From Capri** | 0.0328 | <0.0001 | 0.0926 | 0.0003 | 0 | 0.0008 |
|  | **From Strombolicchio** | 0.0263 | 0.0177 | 0.3311 | 0.000034 | 0 | 0.0002 |
| **Migration into Capri** | **From Palinuro** | 0.0257 | <0.0001 | 0.0735 | 0.00589 | 0 | 0.0155 |
|  | **From Strombolicchio** | 0.0625 | 0.0177 | 0.3311 | 0.00004 | 0 | 0.0001 |
| **Migration into Strombolicchio** | **From Palinuro** | 0.0328 | <0.0001 | 0.0926 | 0.00088 | 0 | 0.0025 |
|  | **From Capri** | 0.0255 | <0.0001 | 0.0732 | 0.0035 | 0 | 0.0084 |

**Supplementary Tab. 2**: Mean (Lower and Upper 95% CI, Confidence Interval) of recent migration rate estimated from BayesAssv3 and of historical migration rate estimated from MIGRATE among 25 individuals (SNPs present in at least 95% individuals).
